# Modelling Divergent Thinking as a Mediator Between Artificial Intelligence Cognitive Stimulation and EFL Learners’ Cognitive Creativity

**DOI:** 10.3390/jintelligence14070139

**Published:** 2026-07-05

**Authors:** Mohammad Hamad Al-khresheh, Suheyla Demirkol Orak, Tariq Elyas, Amal Abdullah Aboras

**Affiliations:** 1 Humanities and Social Research Center, Northern Border University, Arar 73213, Saudi Arabia; 2 Department of Foreign Languages, Faculty of Education, Fırat Unıversity, Elazığ 23100, Türkiye; 3 Department of Modern Languages and Literature, Faculty of Arts & Humanities, King Abdulaziz University, Jeddah 80200, Saudi Arabia; 4 Department of Teaching and Learning, College of Education and Development, Princess Nourah Bint Abdulrahman University, P.O. Box 84428, Riyadh 11671, Saudi Arabia

**Keywords:** AI cognitive stimulation, AI experience, cognitive creativity, divergent thinking, English as a foreign language (EFL), moderated mediation, structural equation modelling (SEM)

## Abstract

Research on AI-supported EFL learning has largely focused on learning outcomes, with limited attention to the cognitive mechanisms underlying creativity and the role of AI experience in shaping this process. This study examined how AI cognitive stimulation relates to EFL learners’ cognitive creativity, as measured by divergent thinking, with attention to the conditioning role of AI experience. Using a quantitative, correlational, cross-sectional design, data were collected from 878 undergraduate EFL learners recruited through stratified cluster sampling across four public universities in Saudi Arabia. Following instrument validation through exploratory and confirmatory factor analysis, structural equation modelling was employed to test direct, indirect, and conditional relationships among the study variables. The findings show that AI cognitive stimulation was strongly associated with divergent thinking (β = 0.52), and divergent thinking was strongly associated with cognitive creativity (β = 0.46). Although AI cognitive stimulation was directly associated with cognitive creativity, this effect was relatively limited. Mediation analysis showed that divergent thinking accounted for the primary pathway linking AI cognitive stimulation to cognitive creativity. Moderated mediation analysis further showed that learners’ duration of AI experience modestly but meaningfully conditioned this indirect pathway without altering its structural form. These results indicate that creative cognition in AI-supported EFL learning depends primarily on learner-controlled ideational processes rather than exposure to AI-generated content alone. The study contributes a process-oriented account of creativity in EFL contexts by specifying the cognitive pattern through which AI-related stimulation is associated with creative capacity and by clarifying how accumulated AI experience conditions this process.

## 1. Introduction

Creativity occupies a central position in contemporary discussions of learning, problem solving, and intellectual development, but educational research has often treated creativity as an outcome rather than a cognitive process. In cognitive terms, creativity involves the generation of original, flexible, and meaningful ideas through processes such as semantic recombination, conceptual restructuring, and adaptive problem solving. Large-scale reviews in education and psychology show that creative performance is frequently reported without systematic attention to the mental operations that give rise to it, resulting in accounts that describe products rather than explain mechanisms (Johnson et al., 2022; Said-Metwaly et al., 2022). This orientation has generated a substantial descriptive literature while leaving core explanatory questions about creative cognition unresolved, particularly in technology-mediated learning environments.

This limitation is visible in research on artificial intelligence in EFL learning. Empirical studies and reviews commonly attribute creativity-related gains to exposure to novel input, alternative formulations, and unconventional linguistic patterns generated by AI systems (Katsarou et al., 2023; Lo et al., 2024; Yang & Kyun, 2022). Such experiences can be viewed as forms of AI cognitive stimulation, referring to the cognitive activation generated through engagement with novel, unexpected, and conceptually varied AI-supported input. While such interpretations appear plausible, they rely on an incomplete explanatory logic. Cognitive research shows that novelty alone does not produce creative thought; learners must reorganise, recombine, and extend information through effortful cognitive activity that exceeds routine language use (Gu et al., 2022; Johnson et al., 2022). When studies attribute creative outcomes directly to AI exposure without specifying how learners cognitively transform AI-generated material, creativity remains an assumed effect rather than a theoretically grounded construct (Shi & Aryadoust, 2024).

Divergent thinking provides a necessary cognitive bridge for addressing this gap. Defined as the capacity to generate multiple, flexible, and original ideas, divergent thinking explains how learners move from exposure to possibility toward creative ideation (Gu et al., 2022; Hubert et al., 2024). In EFL contexts, this process underlies lexical experimentation, alternative syntactic constructions, and conceptual reframing during language production and problem-solving (Johnson et al., 2022; Patterson et al., 2023). Despite its established role in research on verbal creativity, divergent thinking has rarely been posited as a mediating mechanism in empirical models of AI-supported language learning. As a result, existing studies primarily report surface-level associations between AI use and creative outcomes while leaving the intervening cognitive pathway unmodelled (AlTwijri & Alghizzi, 2024).

The implications of this omission extend beyond methodological choice. Treating creativity as a direct consequence of AI engagement risks overstating technological influence while downplaying learners’ cognitive agency. Creativity research consistently demonstrates substantial individual variation in how learners respond to identical environmental stimulation, variation that is explained by differences in semantic memory organisation, executive control, and ideational flexibility (Ovando-Tellez et al., 2022). Models that bypass these processes struggle to account for uneven creative development across learners and contexts, particularly in AI-mediated environments where both stimulation and constraint coexist (Zhai et al., 2024).

The present study addresses this theoretical limitation by modelling divergent thinking as a mediating mechanism between AI cognitive stimulation and cognitive creativity among EFL learners. AI cognitive stimulation is conceptualised as learners’ engagement with novelty, unpredictability, and non-routine associations encountered during AI-supported tasks, rather than exposure to AI output itself. Cognitive creativity is defined as the capacity for original and flexible ideation in language-related problem solving. Within this framework, divergent thinking functions as the cognitive process through which AI-related stimulation is transformed into creative capacity. The model further examines whether this mediated relationship varies according to learners’ duration of AI experience, treating experience as a condition that shapes cognitive engagement rather than as a peripheral background characteristic. Through this process-oriented approach, the study advances an explanatory account of creativity in AI-supported EFL learning that moves beyond outcome-centred descriptions.

## 2. Literature Review

### 2.1. AI Cognitive Stimulation and Creative Language Outcomes in EFL

Research on AI-supported learning increasingly treats artificial intelligence as reshaping the cognitive conditions under which learners engage with language tasks rather than as a tool for efficiency or surface performance (Hubert et al., 2024; Kamalov et al., 2023; Yusuf, 2025). Across educational domains, AI-supported environments are characterised by novelty, variability, and open-ended task structures that shape attentional focus, representational activity, and engagement with meaning-making processes (Almasri, 2024; Gibson et al., 2023). From a cognitive perspective, these conditions are theorised to promote creative performance by expanding opportunities for semantic exploration, conceptual restructuring, and flexible idea generation. In second- and foreign-language learning, these conditions are especially relevant because effective language use depends on the ability to restructure meaning, generate alternative formulations, and manage uncertainty during production and interaction (Yang & Kyun, 2022). Empirical work in higher education indicates that AI functions as a cognitive stimulant when learners actively engage with AI-generated material. Saritepeci and Durak (2024) show that design-based courses require learners to abstract and reframe AI outputs across iterative cycles, whereas students using ChatGPT-4o in flipped research-methods contexts adopt more flexible search strategies and engage in deeper idea manipulation than peers relying on conventional resources (Essel et al., 2023). Mixed-methods evidence further indicates that AI-integrated applications support higher-order engagement under conditions involving diverse perspectives and collaborative problem-solving, whereas rigid task structures restrict such cognitive activity (Lin & Chen, 2024).

At institutional and curricular levels, AI integration has been linked to learning environments structured around open problem spaces and learner agency. Wang et al. (2022) show, through SEM, that institutional AI capability predicts learner outcomes through cognitively meaningful pathways rather than direct performance effects, indicating shifts in task engagement rather than achievement alone. In secondary education, product-based AI pedagogy within AI-focused curricula has been associated with sustained engagement in ill-defined tasks requiring extended reasoning, even when achievement outcomes remain comparable across instructional conditions (Zhan et al., 2022). Reviews of K–12 AI literacy initiatives similarly point to project design and human–computer collaboration as central, framing AI as a stimulus for extended cognitive engagement rather than procedural practice (C. Wu et al., 2022; Yim & Su, 2024). Within EFL research, systematic reviews converge on the view that AI contributes to language learning when it functions as a mediating resource supporting dialogic interaction and learner-controlled transformation of output. Yang and Kyun (2022) argue that such configurations support deeper engagement with language form and meaning than answer-centred applications, a position reinforced by broader syntheses of generative AI that link cognitively productive engagement to task designs requiring ideational variation and learner judgement, as well as risks associated with over-automation. Research on technology-enhanced language learning similarly indicates that instructional innovations can strengthen learner engagement and active participation by promoting meaningful interaction and sustained involvement in learning activities (John, 2024). Evidence from school-based contexts further indicates that AI-personalised content can support sustained textual engagement even when creativity is not explicitly assessed (Jauhiainen & Guerra, 2023).

Research consistently documents conditions under which the use of AI fails to support higher-order cognitive engagement. A systematic review of AI dialogue systems shows that extensive reliance on them encourages heuristic responding and weakens independent evaluation and reasoning processes central to complex cognition (Zhai et al., 2024). Survey-based evidence aligns with this pattern, indicating that greater reliance on AI predicts weaker problem-solving ability despite perceived efficiency gains, reflecting a reduced learner responsibility for cognitive processing (Çela et al., 2024). At the same time, research identifies conditions under which AI-generated novelty supports cognitively demanding outcomes. Doshi and Hauser (2023) describe externalised ideation processes in which AI-provided exemplars lower initial search demands while sustaining exploratory activity. Kim and Maher (2023) report that similarity-based prompting redirects attention toward less salient regions of semantic space, supporting extended conceptual search. Rafner et al. (2023) document how generative systems recombine prior knowledge into unexpected juxtapositions that learners appropriate and revise through active processing, while Miguel-Alonso et al. (2024) report broader problem framing when learners retain responsibility for evaluation and selection. Benvenuti et al. (2023) further suggest that reductions in anxiety and task avoidance in AI-supported environments may indirectly facilitate cognitively demanding engagement by freeing resources for complex processing.

Although AI-supported environments are frequently associated with creative language outcomes in EFL, creativity is rarely modelled as a cognitive process in its own right. Systematic reviews show that creativity-related claims are commonly inferred from performance indicators, leaving the cognitive organisation of creative language use analytically underdeveloped (AlTwijri & Alghizzi, 2024; Katsarou et al., 2023; Yang & Kyun, 2022). Jiang (2022) notes that such outcome-oriented approaches obscure how learners generate, test, and refine ideas during AI-supported tasks, a limitation also identified in reviews of AI-based feedback research that report limited attention to idea construction and revision despite gains in surface quality (Almayez, 2025; Shi & Aryadoust, 2024). Empirical EFL studies nonetheless indicate that creative cognition is more likely when learners actively reorganise and evaluate AI-generated input. Dialogic AI environments support negotiation of meaning and discursive flexibility when tasks prioritise authentic interaction over procedural practice (Alshumaimeri & Alshememry, 2024; Jiang, 2022), while AI-supported speaking research shows that performance gains co-occur with stronger self-regulatory control and motivated engagement, cognitive states linked to exploratory language use (Qiao & Zhao, 2023; Wei, 2023). In writing instruction, classroom-based studies report stronger cohesion and interpretive engagement when learners critically transform AI-generated material rather than reproduce it verbatim (Tseng & Lin, 2024). Individual-difference research reinforces this pattern, indicating that learners with higher metacognitive awareness use AI to stimulate idea development and planning. In contrast, weaker regulation is associated with uncritical replication and constrained creativity (Abdelhalim, 2024).

Despite these contributions, the internal cognitive processes that link AI-driven stimulation to creative language outcomes remain poorly specified. Neurocomputational accounts caution that shifts in semantic association and ideational breadth are frequently assumed rather than empirically traced in studies of AI-supported learning (Khalil & Moustafa, 2022). Empirical research rarely examines how learners move between expansive idea generation and evaluative refinement during AI-supported tasks, or how curiosity-triggering prompts translate into sustained exploratory behaviour over time (Wingström et al., 2022; Zirar, 2023). Similar patterns of cognitively productive and counterproductive AI use have been reported across student, teacher, school, and professional training contexts, including research on generative language models, collaborative design, AI literacy, and pre-service teacher engagement (Atkinson & Barker, 2023; Ayanwale et al., 2024; Gordon et al., 2024; Habib et al., 2023; Hubert et al., 2024; Kamalov et al., 2023; Y. Liu et al., 2023; Marrone et al., 2022).

### 2.2. Divergent Thinking as a Cognitive Mechanism in Language Learning

Divergent thinking occupies a central position in cognitive accounts of creativity as a process that enables the generation of multiple, flexible, and non-redundant ideas rather than convergence on a single solution (Gu et al., 2022; Hubert et al., 2024; Wigert et al., 2022). Cognitive theories of creativity generally view divergent thinking as a mechanism by which individuals broaden their semantic search, access remote associations, and generate novel conceptual combinations that support creative performance. Within language learning, this process underpins creative language use by allowing learners to reconfigure meaning, construct alternative expressions, and navigate communicative uncertainty during production and interaction. Creative performance in an additional language, therefore, rests less on efficiency or accuracy than on the capacity to search, extend, and reorganise semantic representations across linguistic resources (Yang & Kyun, 2022).

Cognitive research characterises divergent thinking through operations such as fluency, flexibility, originality, and elaboration, which jointly index learners’ capacity to traverse semantic space rather than replicate conventional patterns (Gu et al., 2022; Hubert et al., 2024). Studies of verbal creativity using distributional semantic modelling show that creative texts tend to integrate semantically distant concepts, a property captured by measures of semantic divergence that predict human judgments of creativity in narrative and verbal tasks, including those produced by L2 speakers (Johnson et al., 2022; Patterson et al., 2023). These findings indicate that creative language use depends on learners’ ability to activate and combine remote associations within multilingual semantic networks rather than rely on habitual formulations.

This perspective also aligns with broader research on creativity and intelligence, in which divergent thinking is viewed as a product of interactions among associative processing, semantic memory organisation, and executive control. Associative theories of creativity suggest that creative individuals are better able to access and combine remote concepts, enabling them to move beyond conventional thought patterns and generate more original ideas (Beaty & Kenett, 2023). Recent research on semantic-memory networks further indicates that differences in the organisation and flexibility of conceptual knowledge are closely related to creative performance, with more interconnected and adaptable networks supporting idea generation across a range of contexts (Li et al., 2024). Similar conclusions have been reported in studies of verbal creativity, where the integration of semantically distant concepts has been linked to higher levels of originality and creative expression (Johnson et al., 2022; Patterson et al., 2023). Executive processes, including cognitive flexibility, attentional regulation, and evaluative monitoring, also contribute to the generation and refinement of ideas, helping individuals move between idea production and idea selection during creative tasks (Said-Metwaly et al., 2022). Viewed from this perspective, divergent thinking is a core cognitive process that links intelligence-related cognitive resources to creative performance across domains, including language learning.

EFL research provides converging evidence that instructional conditions that support divergent thinking are associated with greater generative language use. Creative writing tasks structured around divergent heuristics, such as SCAMPER, support conceptual expansion and the production of problem-oriented language (Şenel, 2018). Experimental work targeting divergent operations through schema violation, random association, and conceptual expansion demonstrates measurable gains in verbal divergent thinking, whereas unguided ideation yields weaker cognitive effects (Gu et al., 2022). Classroom-based studies further show that inquiry-oriented, reflective, and translanguaging pedagogies facilitate flexible meaning-making and creative engagement across speaking and writing, positioning creativity as a cognitively scaffolded outcome rather than an incidental instructional by-product (Greenier et al., 2023; Kumar et al., 2023; Riswanto et al., 2022; Yuzlu & Dikilitaş, 2021).

Within AI-supported learning contexts, divergent thinking cannot be assumed to follow automatically from exposure to generative systems. Although large language models frequently produce outputs rated highly for originality and elaboration, their generative behaviour is driven by statistical patterning that differs fundamentally from human cognitive processing (Hubert et al., 2024; Magee et al., 2022; Mitchell & Krakauer, 2022). From a cognitive creativity perspective, creative performance depends on learners actively engaging in semantic exploration, evaluative judgement, and the generation of alternative possibilities rather than passively receiving externally generated content. Further work on prompting strategies and multi-agent reasoning shows that, unless instructional designs deliberately require learners to generate, compare, and evaluate alternatives, generative systems tend to stabilise around convergent solution paths that restrict conceptual breadth (Said-Metwaly et al., 2022). This instructional configuration risks shifting responsibility for ideation from learners to the system, thereby weakening engagement with the cognitive operations that support creative language use. Empirical studies reinforce this concern by demonstrating that AI-mediated instruction can either support or constrain divergent thinking depending on task design: ChatGPT-supported flipped learning strengthens creative and reflective thinking when learners critically interrogate and revise AI-generated content, whereas other implementations report reduced creative engagement under conditions of cognitive overload or excessive automation (Essel et al., 2023). Evidence from EFL pedagogy converges on a similar conclusion, showing that practices such as process writing, peer assessment, reflective evaluation, and inquiry-based learning foster higher-order cognition precisely because they require learners to generate, assess, and refine multiple possibilities rather than consume pre-constructed responses (Kumar et al., 2023; Riswanto et al., 2022).

Across creativity research, cognitive psychology, and EFL pedagogy, divergent thinking is treated as a distinct, trainable cognitive mechanism rather than a proxy for proficiency or exposure. Meta-analytic and psychometric work shows that divergent thinking predicts creative achievement moderately and independently of general ability, reinforcing the need for explicit instructional attention to this process (Johnson et al., 2022; Patterson et al., 2023; Said-Metwaly et al., 2022). In AI-supported EFL contexts, creative outcomes therefore depend on whether learners remain responsible for semantic exploration, idea generation, and evaluative selection. Without explicit modelling of divergent thinking at the task and feedback levels, AI-supported instruction risks replacing the very cognitive operations that underlie creative language use.

### 2.3. Divergent Thinking as a Mediator Between AI Cognitive Stimulation and Cognitive Creativity

Evidence from AI-supported EFL learning suggests that creative language outcomes cannot be explained by direct exposure to AI. Essel et al. (2023) report that learners using comparable AI tools exhibit substantial variation in originality and flexibility, despite similar levels of access and task design. Parallel findings in AI-supported speaking indicate that performance gains often co-occur with changes in self-regulation and strategic engagement rather than with AI use per se (Qiao & Zhao, 2023). Longitudinal modelling further shows that higher-order outcomes in AI-assisted writing are predicted by learners’ cognitive engagement patterns over time rather than by tool affordances alone (Shen & Teng, 2024). Creativity research provides a consistent explanation for this pattern: external stimulation expands opportunity spaces, but novelty in output depends on how learners internally reorganise and transform available material (Bai et al., 2021). These converging findings weaken direct-effect accounts and motivate a process-based explanation.

Divergent thinking offers such an explanation by specifying the cognitive operation that links AI stimulation to creative language use. Gu et al. (2022) frame divergent thinking as the regulation of ideational breadth, flexibility, and originality through associative search and executive control. In verbal domains, this involves generating alternative expressions, shifting semantic frames, and recombining meanings rather than refining a single response (Bai et al., 2021). Ovando-Tellez et al. (2022) demonstrate that creative language performance is associated with patterns of semantic memory search that allow access to more distant conceptual regions. Such findings suggest that creative outcomes depend not merely on exposure to novel information but on learners’ ability to navigate, reorganise, and integrate semantic representations during ideation. Neurocognitive evidence further indicates that creative performance depends on internal network dynamics that govern whether stimulation broadens or constrains semantic exploration, independent of task input (Shofty et al., 2022). Consequently, in AI-supported EFL contexts, generative systems function as sources of linguistic material, whereas divergent thinking determines whether that material is transformed into novel language or absorbed through convergent imitation. Modelling divergent thinking as a mediator explicitly captures this cognitive transformation.

The strength of this mediating pathway varies with learners’ duration of AI experience. Studies of AI adoption in EFL show that early-stage users devote substantial cognitive resources to managing the tool and prioritising correctness, thereby limiting engagement in exploratory processing (C. Liu et al., 2021). H. Wu et al. (2024) similarly report that initial AI use is shaped by usability concerns and uncertainty, constraining strategic experimentation. With increased experience, learners develop greater AI literacy and confidence, allowing them to treat AI outputs as provisional material to be questioned, varied, and recombined rather than accepted wholesale. Evidence from university-level AI-supported instruction indicates that such strategic use is associated with greater gains in creative and reflective thinking (Essel et al., 2023; Song & Song, 2023). Shen and Teng (2024) further report that, over time, higher-order writing outcomes increasingly depend on learners’ self-directed engagement rather than on AI assistance per se. At the same time, systematic reviews caution that prolonged, uncritical reliance on AI dialogue systems can encourage heuristic acceptance and weaken independent reasoning, thereby reducing divergent thinking despite extensive exposure (Zhai et al., 2024). Treating AI experience duration as a conditioning factor therefore accounts for variability in creative outcomes. It supports a moderated mediation model in which AI cognitive stimulation indirectly influences creativity through divergent thinking, under conditions shaped by learners’ evolving interactions with AI.

### 2.4. Research Gap and Proposed Hypotheses

Research on AI-supported EFL learning has expanded rapidly, documenting associations between AI use and learner outcomes, including performance, engagement, motivation, and indicators of creativity. Large-scale reviews and syntheses show that empirical work in this area continues to prioritise measurable outcomes linked to achievement, affect, or behavioural engagement, while offering limited examination of the cognitive processes activated during AI-mediated language use (AlTwijri & Alghizzi, 2024; Katsarou et al., 2023; Lo et al., 2024; Yang & Kyun, 2022). Where creativity is reported, it is often operationalised as a downstream outcome inferred from task performance or product quality, rather than as a cognitive phenomenon requiring process-level explanation. Consequently, existing evidence remains largely descriptive of what changes under AI-supported conditions but is theoretically thin on how those changes occur at the cognitive level.

A related limitation concerns the treatment of learner cognition within AI–creativity research in EFL. Creativity-related effects are frequently attributed directly to AI exposure, task affordances, or instructional configurations, with little effort to specify the internal cognitive processes through which AI-supported input is transformed into original language use. Reviews of AI-assisted writing, speaking, and dialogue systems note that studies rarely model intermediate cognitive variables, instead assuming that access to rich input or feedback translates linearly into creative outcomes (Shi & Aryadoust, 2024; Song & Song, 2023; Zhai et al., 2024). Creativity research, by contrast, consistently identifies divergent thinking as a central cognitive operation underlying creative language production, particularly in verbal and second-language contexts (Gu et al., 2022; Johnson et al., 2022; Said-Metwaly et al., 2022). The absence of divergent thinking as an explicitly modelled mechanism, therefore, represents a significant conceptual omission in AI-supported EFL research.

A further gap concerns learners’ experience with AI systems. The duration of AI use is typically reported as background information or treated as a control variable, rather than conceptualised as a condition that shapes how learners cognitively engage with AI-generated output. Empirical studies indicate that learners’ interaction patterns, evaluative stance, and reliance on AI outputs evolve with continued use, influencing whether AI functions as a cognitive partner or a shortcut for task completion (Y. Liu et al., 2023; Shen & Teng, 2024; H. Wu et al., 2024). Systematic reviews similarly caution that prolonged or uncritical reliance on AI dialogue systems can alter cognitive engagement, thereby suppressing independent reasoning and generative processing (Zhai et al., 2024). Despite this evidence, to the best of our knowledge, no EFL study has examined whether the cognitive pathway linking AI-related stimulation to creative outcomes varies with learners’ accumulated AI experience.

These limitations indicate the absence of a process-oriented model that can explain AI-driven creativity in EFL learning. In particular, no empirical study has tested whether divergent thinking functions as the mechanism through which AI cognitive stimulation relates to cognitive creativity, nor whether this indirect relationship is contingent on learners’ duration of AI experience. Addressing this gap, the present study advances and tests a moderated mediation model grounded in cognitive creativity research and AI-supported language learning. The study, therefore, addresses the following research question:

To what extent does AI cognitive stimulation relate to cognitive creativity among EFL learners directly and indirectly through divergent thinking, and how does learners’ duration of AI experience condition this indirect relationship?

Based on the theoretical synthesis developed in the preceding sections, the following hypotheses are proposed:

**H1.**  *AI cognitive stimulation positively predicts divergent thinking among EFL learners.*

**H2.**  *Divergent thinking positively predicts cognitive creativity in EFL learning contexts.*

**H3.**  *AI cognitive stimulation positively predicts cognitive creativity.*

**H4.**  *Divergent thinking mediates the relationship between AI cognitive stimulation and cognitive creativity.*

**H5.**  *The indirect effect of AI cognitive stimulation on cognitive creativity through divergent thinking is expected to demonstrate a stronger standardised association than the direct effect based on bootstrapped SEM estimates.*

**H6.**  *The indirect effect of AI cognitive stimulation on cognitive creativity via divergent thinking varies according to learners’ duration of AI experience, such that the mediated effect is stronger among learners with longer AI experience.*

**Figure 1:**
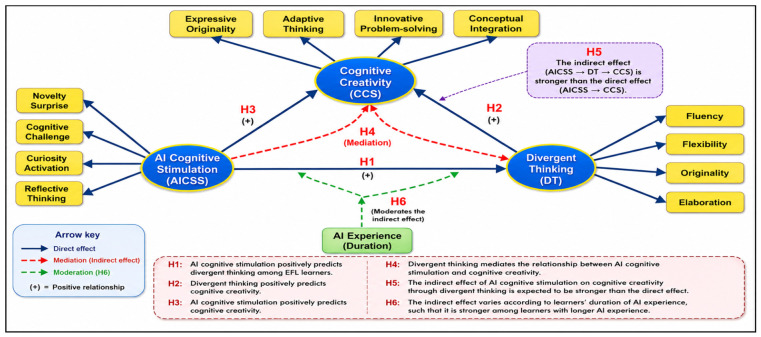
Proposed Conceptual Model of AI Cognitive Stimulation, Divergent Thinking, and Cognitive Creativity.

To consolidate the theoretical arguments and hypotheses, this study proposes a conceptual model specifying the cognitive pathways linking AI engagement and creativity in EFL learning. The model frames AI cognitive stimulation as a source of non-routine linguistic and conceptual input, divergent thinking as the central mechanism governing ideational generation and transformation, and cognitive creativity as the capacity for flexible and original language-related problem solving. The model distinguishes direct associations from indirect effects mediated by divergent thinking and accounts for variation in learners’ accumulated AI experience. Figure 1 depicts the hypothesised relationships and the moderated mediation structure.

## 3. Research Methods

### 3.1. Research Design

The study adopted a quantitative, correlational research design grounded in SEM to examine the relationships among AI cognitive stimulation, divergent thinking, cognitive creativity, and learners’ duration of AI experience in EFL learning. This design was selected to allow simultaneous examination of direct, indirect, and conditional relationships among latent constructs within a theoretically specified model (Barrett, 2007). A moderated mediation framework was applied to capture both the cognitive process linking AI-related stimulation to creative outcomes and the conditioning role of accumulated AI experience. Data were collected at a single point in time using validated self-report instruments aligned with the study’s process-oriented conceptualisation of creativity, focusing on internal cognitive engagement rather than on task performance or frequency of technology use. This design supported rigorous testing of the hypothesised pathways while remaining appropriate for large-scale investigation within authentic university EFL contexts.

### 3.2. Participant Recruitment

Participants were recruited from four public universities in Saudi Arabia offering undergraduate EFL programmes. The study was therefore situated within the Saudi EFL higher-education context. The study employed a stratified cluster sampling strategy, selected to balance institutional representation with the practical constraints of classroom-based data collection. Intact classes within English-related programmes served as sampling clusters. Stratification was applied at the institutional level and by year of study to reduce disproportionate representation across universities and academic stages. Individual-level random sampling was not implemented because participation occurred within preexisting instructional groups.

A total of 932 undergraduate students participated in the study. Following data screening, responses with substantial missing data were excluded, leaving 878 valid cases for analysis. The final sample size was appropriate for latent variable modelling involving multiple constructs and indirect pathways. Methodological guidance for SEM indicates that samples exceeding 500 participants provide adequate statistical power for detecting medium-sized direct and indirect effects in mediation and conditional process models. The retained sample exceeded this threshold, supporting stable parameter estimation.

The demographic characteristics of the participants are summarised in Table 1. The sample comprised 302 male students (34.4%) and 576 female students (65.6%). Most participants were aged 21–23 years (44.7%), followed by 18–20 years (27.1%), 24–26 years (19.1%), and 27 years or older (9.1%). Academic-level representation was distributed across first-year (24.4%), second-year (29.2%), third-year (27.1%), and fourth-year or higher students (19.4%). Self-rated English proficiency indicated that most participants identified as intermediate learners (53.8%), with smaller proportions reporting advanced (25.1%) or beginner levels (21.2%).

Engagement with AI for English learning varied across the sample, with participants reporting AI use ranging from less than 6 months to more than 2 years. This variation supported examination of experience-related differences in cognitive engagement within the proposed model.

### 3.3. Instruments

Data were collected through a structured self-report questionnaire comprising four components: demographic information, the AI Cognitive Stimulation Scale (AICSS), the Divergent Thinking Scale (DTS), and the Cognitive Creativity Scale (CCS). All scale items were framed in terms of learners’ engagement in AI-supported English learning tasks, with attention directed toward cognitive processes rather than to the frequency of tool use or perceived instructional effectiveness. Responses to all scale items were recorded on a five-point Likert continuum ranging from ‘*strongly disagree* to *strongly agree*’ (see Supplementary S1—Supplementary Materials).

Demographic and Background Information: Participants reported their gender, age group, and university level. Self-rated English proficiency was measured using CEFR-aligned categories (A2–C1). Patterns of AI engagement were captured by the frequency of AI tool use and the duration of AI experience. Duration of AI experience, measured categorically as less than six months, six to 12 months, 12 to 24 months, or more than 2 years, was specified as the conditioning variable in the moderated mediation model.

AICSS: AI cognitive stimulation was measured using a 24-item scale developed to operationalise learners’ cognitive engagement with AI-supported language tasks. Scale development was guided by theoretical accounts that conceptualise AI as shaping the cognitive conditions of learning through exposure to non-routine input, conceptual variation, and reflective engagement rather than through direct performance effects (Gibson et al., 2023). Items were organised into four dimensions: Novelty and Surprise, Cognitive Challenge, Curiosity Activation, and Reflective Thinking. These dimensions capture learners’ engagement with unfamiliar perspectives, evaluative comparison of alternatives, inquiry-oriented exploration, and self-reflective regulation during interaction with AI-generated content. The scale was designed to reflect cognitively productive AI engagement as learner-mediated activity, consistent with EFL-focused syntheses that distinguish reflective and evaluative AI use from procedural reliance (Jiang, 2022; Shi & Aryadoust, 2024). Participants reported engagement with a range of generative AI and AI-assisted language tools commonly used in EFL learning contexts, including conversational AI systems, AI-supported writing tools, and idea-generation platforms. AI-supported activities primarily involved writing, brainstorming, reading support, paraphrasing, language practice, and interactive dialogue tasks. The study focused on learners’ perceived cognitive engagement during AI-supported language activities rather than on the effectiveness of any single AI platform or instructional application.

DTS: Divergent thinking was assessed using a 24-item scale designed to measure generative ideation in English language tasks. The scale was constructed in line with cognitive models that define divergent thinking as a multi-component process involving fluency, flexibility, originality, and elaboration (Gu et al., 2022; Wigert et al., 2022). The four dimensions capture learners’ capacity to generate multiple ideas, shift perspectives, produce uncommon expressions, and develop ideas through expansion and structuring. The item wording was adapted for English learning contexts to reflect evidence that divergent thinking functions as a domain-relevant cognitive process in verbal creativity rather than as a general creative disposition (Johnson et al., 2022; Said-Metwaly et al., 2022).

CCS: Cognitive creativity was measured using a 24-item scale assessing learners’ perceived capacity for creative cognition in English language use. Scale development drew on research that positions creativity as a function of internal cognitive organisation, semantic recombination, and executive regulation rather than surface novelty or task performance (Rafner et al., 2023). The scale comprises four dimensions: Expressive Originality, Adaptive Thinking, Innovative Problem-Solving, and Conceptual Integration. These dimensions capture original expression, flexible adaptation to task demands, inventive handling of communicative challenges, and integration of ideas across conceptual domains. Item content reflects EFL research showing that creative language use depends on learners’ capacity to reorganise meaning and apply ideas flexibly across contexts (Greenier et al., 2023; Yang & Kyun, 2022). The scale was designed to assess learners’ self-perceived capacity for creative cognition rather than objectively observed creative performance. Accordingly, the construct reflects participants’ evaluations of their own creative cognitive tendencies in English-language use, and the findings should be interpreted as indicators of perceived creative capacity rather than as direct measures of creative output.

### 3.4. Instruments Validation Procedures

The validation of the research instruments followed a structured, multi-stage procedure designed to establish content adequacy and factorial stability prior to the main study. Given that the study employed newly developed measures aligned with a process-oriented model, validation procedures were implemented independently from hypothesis testing and structural modelling.

An initial pool of items for the AICSS, DTS, and CCS underwent expert review. Two reviewers with backgrounds in applied linguistics and educational psychology evaluated items for conceptual relevance, clarity, and alignment with the theoretical definitions underpinning each construct. Based on this review, items identified as ambiguous, overlapping, or insufficiently representative of the targeted cognitive processes were revised or removed. This stage aimed to secure adequate conceptual coverage across dimensions while maintaining scale coherence. The preliminary item pools subjected to exploratory factor analysis comprised 24 items each for AICSS, DTS, and CCS. Item generation was guided through theoretical synthesis of prior literature on AI-supported learning, divergent thinking, creativity, and cognitive engagement in EFL contexts. Items were developed through a combination of theoretical derivation and adaptation of construct definitions reported in previous creativity and AI-related research. The expert reviewers possessed expertise in language learning, psychometrics, and cognitive creativity research. In addition to evaluating conceptual relevance and dimensional alignment, the reviewers examined wording clarity, redundancy, and potential overlap across dimensions. Based on their recommendations, several items were revised for linguistic precision and conceptual specificity, while conceptually overlapping or ambiguous items were removed prior to pilot testing. Following exploratory factor analysis, items demonstrating weak primary factor loadings or elevated cross-loadings were excluded prior to confirmatory factor analysis. Items with primary factor loadings below 0.50 or cross-loadings exceeding 0.30 were considered for removal. This refinement process yielded the final set of retained indicators used in the main study.

A pilot study was conducted with 414 EFL learners drawn from the same population as the main study. To support robust construct validation and to prevent overfitting, the pilot sample was randomly divided into two independent subsamples. One subsample (n = 207) was used exclusively for exploratory factor analysis (EFA), while the second subsample (n = 207) was reserved for confirmatory factor analysis (CFA). This split-sample procedure was adopted to allow independent examination of the latent structure and to strengthen the credibility of the measurement model.

EFA was conducted on the first subsample to examine the underlying dimensional structure of each instrument. The procedure aimed to assess whether items were grouped according to the theoretically proposed dimensions. EFA was conducted using Principal Axis Factoring with Promax rotation. The oblique rotation procedure was selected to allow correlations among the latent dimensions, consistent with the theoretical expectation that the factors would be related. Decisions regarding item retention were based on factor interpretability, dimensional coherence, alignment with construct definitions, magnitude of primary factor loadings, and the absence of problematic cross-loadings. CFA was conducted on the second subsample to evaluate the factor structures identified during the exploratory phase. CFA was used solely to verify whether the hypothesised measurement models were supported by the data when tested on an independent sample. CFA and subsequent SEM were estimated using Maximum Likelihood (ML) estimation in AMOS. ML estimation was considered appropriate given the large sample size and the data’s acceptable distributional properties. Each construct was specified as a multidimensional latent variable, with items linked to their respective factors as defined theoretically. Item retention decisions during EFA were based on factorial interpretability and psychometric adequacy. Items with primary factor loadings below 0.50 or cross-loadings exceeding 0.30 were considered for removal. The refinement process was conducted iteratively to improve dimensional clarity and reduce overlap across factors. Only items demonstrating satisfactory factorial concentration and conceptual coherence were retained for confirmatory analysis. To examine potential common method bias associated with self-report measures, Harman’s single-factor test was conducted using unrotated exploratory factor analysis across all measurement items. The analysis indicated that the first unrotated factor accounted for 34.2% of the total variance, which remained below the commonly referenced threshold of 50%. This finding suggests that common method bias was unlikely to substantially affect the observed relationships among the study variables.

Procedures for assessing internal consistency and construct validity were incorporated at the measurement level. Internal consistency was examined at the construct and dimension levels, and convergent and discriminant validity were assessed as part of the measurement model evaluation. These procedures were implemented to confirm that each scale captured a distinct cognitive construct consistent with its theoretical specification. To support subsequent model testing stratified by AI experience duration, procedures to examine measurement equivalence across groups were incorporated into instrument validation. This step aimed to verify that the constructs were represented consistently across experience categories prior to structural modelling (see the factor analysis findings in the next section).

### 3.5. Data Collection and Ethical Considerations

Data were collected during the first term of the 2025–2026 academic year over a period of two months using a structured self-report questionnaire administered to undergraduate EFL learners at four public universities. The questionnaire was distributed through Qualtrics, with access provided during regular teaching periods to students enrolled in the selected intact classes. Although participants were recruited through intact classes, the classes served solely as a practical mechanism for participant access and data collection rather than as instructional or treatment groups. Participants completed the questionnaire independently, and the constructs under investigation reflected individual cognitive perceptions and experiences rather than shared classroom characteristics. Consequently, the analyses were conducted at the individual level of observation. Participants completed the survey individually and were instructed to base their responses on their experiences with AI-supported English-learning tasks, focusing on cognitive engagement rather than general attitudes toward technology. Prior to participation, students received a written explanation of the study’s purpose, the voluntary nature of participation, and the academic use of the data, and they provided informed consent electronically via survey entry. No personally identifying information was collected, participation carried no academic consequences, and no incentives were offered. Ethical approval was obtained from the relevant institutional ethics committee at Northern Border University, and all procedures complied with institutional ethical requirements and established principles governing confidentiality, voluntary participation, and the responsible handling of research data.

### 3.6. Data Analysis

Data analysis was conducted in several stages using SPSS (Version 28) and AMOS (Version 28). Preliminary analyses in SPSS addressed data screening, missing values, distributional properties, and collinearity to confirm the suitability of the data for latent variable modelling. Instrument validation followed a split-sample procedure: EFA was performed in SPSS on the first subsample to examine the dimensional structure, and CFA was conducted in AMOS on an independent subsample to verify the measurement models. Internal consistency, composite reliability, average variance extracted, and discriminant validity were examined at the measurement level. SEM was then implemented in AMOS to test the hypothesised direct and indirect relationships among AI cognitive stimulation, divergent thinking, and cognitive creativity. The structural model was specified a priori based on the theoretical framework and hypotheses developed from the literature. Alternative structural specifications were not estimated or compared because the objective was to evaluate the theoretically derived model rather than to identify the best-fitting post hoc structure. Moderated mediation was examined using interaction terms involving duration of AI experience, and conditional indirect effects were evaluated using bias-corrected bootstrap confidence intervals based on 5000 resamples. The moderation effects were estimated using observed product-term interactions (AICSS × AI Experience and DTS × AI Experience), consistent with a PROCESS-style approach to moderated mediation implemented within the SEM framework. Model adequacy was assessed using established fit indices appropriate for large-sample analysis of Likert-type data. To assess potential common-method bias associated with the use of self-report instruments administered in a single session, two diagnostic procedures were conducted. First, Harman’s single-factor test was performed using unrotated exploratory factor analysis across all retained measurement items. The first unrotated factor accounted for 34.2% of the total variance, remaining below the commonly referenced 50% threshold. Second, a common latent factor model was estimated in AMOS by adding a latent method factor to the retained indicators. Comparisons between the original CFA model and the common latent factor model showed that changes in standardised factor loadings remained below 0.20, indicating that method-related variance did not substantially alter the measurement estimates. These results suggest that common-method bias was unlikely to materially affect the structural findings.

## 4. Findings

### 4.1. Exploratory Factor Analysis

EFA was conducted to examine the latent structure of the three instruments prior to confirmatory modelling. Separate EFAs were performed for AICSS, DTS, and CCS using the first pilot subsample (n = 207). The analyses were undertaken to assess sampling adequacy and factorability before evaluating dimensional structure. As reported in Table 2, the Kaiser–Meyer–Olkin (KMO) measures indicated excellent sampling adequacy for all three scales, with values ranging from 0.92 to 0.94. Bartlett’s tests of sphericity were statistically significant for the AICSS, DTS, and CCS (*p* < .001), confirming that the correlation matrices were suitable for factor extraction. These results indicate that the item sets shared sufficient common variance to support EFA and to justify proceeding with the examination of the underlying factor structures for each construct.

Before examining item–factor relationships, the distribution of variance across extracted components was inspected to determine the latent structure of the AI Cognitive Stimulation Scale. As reported in Table 3, the first component produced an eigenvalue of 8.21, accounting for 34.21% of the total variance. The second component yielded an eigenvalue of 3.42, accounting for an additional 14.25%, bringing the cumulative variance explained to 48.46%. The third and fourth components had eigenvalues of 2.11 and 1.56, accounting for 8.79% and 6.50% of the variance, respectively. These four components jointly accounted for 63.75% of the total variance. All subsequent components had eigenvalues below 1.00 and individually contributed only marginal proportions of variance, indicating limited substantive relevance. The concentration of explained variance within the first four components supported retention of a four-factor structure for subsequent factor interpretation.

Following the assessment of sampling adequacy, attention shifted to the DTS’s variance structure to determine its underlying dimensionality. As reported in Table 4, the first component yielded an eigenvalue of 7.64, accounting for 31.83% of the total variance. The second component accounted for 13.58% of the variance (eigenvalue = 3.26), bringing the cumulative variance explained to 45.41%. The third and fourth components explained 9.75% (eigenvalue = 2.34) and 7.58% (eigenvalue = 1.82), respectively, resulting in a cumulative variance of 62.74%. Subsequent components had eigenvalues below unity and accounted for only limited additional variance, supporting retention of a four-factor structure for further analysis.

Building on the variance patterns observed for the preceding scales, the dimensional structure of the CCS was examined by inspecting the explained variance. As reported in Table 5, the first component yielded an eigenvalue of 7.92, accounting for 33.00% of the total variance. The second component accounted for 13.25% of the variance (eigenvalue = 3.18), bringing the cumulative variance explained to 46.25%. The third and fourth components recorded eigenvalues of 2.27 and 1.68, explaining 9.46% and 7.00% of the variance, respectively. These four components jointly accounted for 62.71% of the total variance. Subsequent components had eigenvalues below 1.00 and contributed little additional variance, supporting the retention of a four-factor structure for further analysis of cognitive creativity.

**Figure 2:**
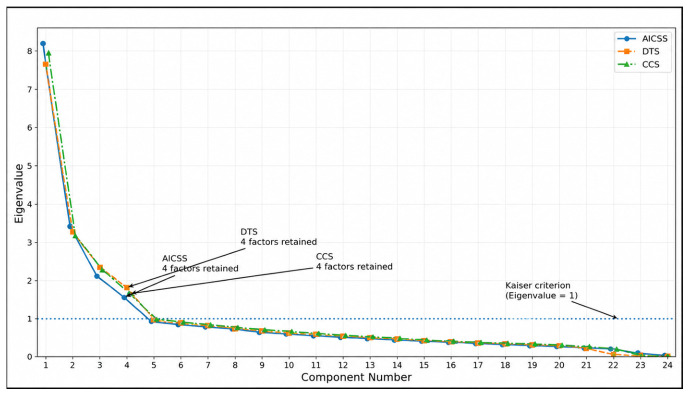
Scree plot for AICSS, DTS and CCS.

Following inspection of variance distributions, scree plots were examined to corroborate factor retention decisions across AICSS, DTS, and CCS. As shown in Figure 2, all three scales exhibited steep declines in eigenvalues across the first four components, followed by evident flattening thereafter. For AICSS, eigenvalues declined from 8.21 for the first component to 1.56 for the fourth, with subsequent components below 1.00. A similar pattern was observed for DTS, with eigenvalues decreasing from 7.64 to 1.82 across the first four components, and for CCS, with eigenvalues declining from 7.92 to 1.68. In each case, components beyond the fourth were associated with eigenvalues below unity and minimal incremental variance. The convergence between scree plot inspection and variance analysis supports retention of four-factor structures for AICSS, DTS, and CCS prior to confirmatory modelling.

The rotated factor solution for AICSS was examined to evaluate item alignment with the proposed four-dimensional structure. As shown in Table 6, items representing Novelty and Surprise loaded on the first factor with coefficients between 0.73 and 0.84, while Cognitive Challenge items loaded on the second factor with values ranging from 0.71 to 0.86. The third factor, corresponding to Curiosity Activation, showed primary loadings between 0.71 and 0.82, and Reflective Thinking items loaded on the fourth factor with coefficients ranging from 0.69 to 0.88. Several items (e.g., NV4, NV6, CC4, CC6, CA3–CA6, and RT5) demonstrated weak primary loadings or elevated cross-loadings during EFA and were excluded prior to confirmatory factor analysis, indicating limited factorial specificity. Overall, the rotated solution showed satisfactory separation across the four dimensions and was consistent with the intended construct structure.

Inspection of the rotated solution for DTS focused on the degree to which items clustered around the intended dimensions. As presented in Table 7, Fluency items showed their strongest associations with the first factor, with loadings between 0.72 and 0.86. The second factor was defined by Flexibility items, which loaded between 0.76 and 0.88. Items intended to capture Originality aligned with the third factor, exhibiting primary loadings from 0.73 to 0.84, while Elaboration items were associated with the fourth factor, with coefficients ranging from 0.78 to 0.87. Several items (e.g., FL3, FL5, FLEX5, FLEX6, ORIG2, ORIG3, ORIG6, ELAB2, ELAB5, and ELAB6) demonstrated weak primary loadings or elevated cross-loadings during EFA and were excluded prior to confirmatory factor analysis, indicating weaker factorial concentration. Despite this, the overall loading pattern supported a four-factor representation of divergent thinking consistent with the scale’s theoretical specification.

The rotated factor matrix for CCS showed clear separation across the four intended dimensions. As reported in Table 8, Expressive Originality items loaded on the first factor with coefficients between 0.72 and 0.87, Adaptive Thinking items defined the second factor with loadings from 0.77 to 0.86, Innovative Problem-Solving items aligned with the third factor with values ranging from 0.74 to 0.85, and Conceptual Integration items loaded on the fourth factor with coefficients between 0.79 and 0.88. Several items (e.g., EO3, EO5, AT4–AT6, IPS1, IPS3, IPS5, and CI2, CI4, and CI6) demonstrated weak primary loadings or elevated cross-loadings during EFA and were excluded prior to confirmatory factor analysis, indicating reduced dimensional focus rather than overlap. Overall, the pattern of loadings supported the proposed four-factor structure of cognitive creativity.

### 4.2. Confirmatory Factor Analysis

Following the establishment of factorial structure through exploratory analysis, CFA was undertaken to assess whether the proposed measurement models for AICSS, DTS, and CCS were supported when tested on an independent sample. As reported in Table 9, all three models demonstrated satisfactory fit. The AICSS model yielded a χ^2^ statistic of 214.63 with 98 degrees of freedom (χ^2^/df = 2.19), along with fit indices of CFI = 0.964, TLI = 0.958, RMSEA = 0.052, and SRMR = 0.041. For DTS, the model produced a χ^2^ of 198.47 with 94 degrees of freedom (χ^2^/df = 2.11), with corresponding values of CFI = 0.961, TLI = 0.953, RMSEA = 0.049, and SRMR = 0.043. The CCS model showed a χ^2^ of 186.92 with 88 degrees of freedom (χ^2^/df = 2.12), accompanied by CFI = 0.968, TLI = 0.961, RMSEA = 0.047, and SRMR = 0.039. The consistency of these indices across models indicates that the hypothesised factor structures were adequately represented and appropriate for subsequent structural modelling.

**Figure 3:**
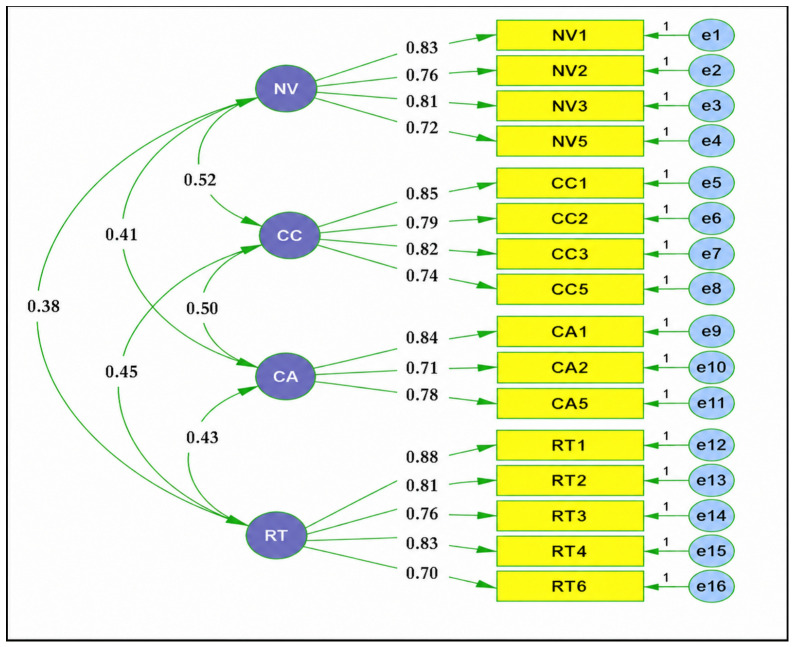
CFA for AICSS.

The confirmatory factor model for AICSS is presented in Figure 3 and corresponds to the four-dimensional structure identified through exploratory analysis. Standardised factor loadings were substantial across all retained indicators, ranging from 0.72 to 0.83 for Novelty and Surprise, 0.74 to 0.85 for Cognitive Challenge, 0.71 to 0.84 for Curiosity Activation, and 0.70 to 0.88 for Reflective Thinking. Inter-factor correlations were moderate, with coefficients ranging from 0.38 to 0.52, indicating conceptual relatedness while preserving dimensional distinctiveness within the construct of AI cognitive stimulation. The configuration of loadings and latent correlations supports the adequacy of the specified measurement model and is consistent with the scale’s underlying theoretical structure.

Following confirmation of the measurement structure, a combined higher-order CFA was also estimated to assess the measurement structure of AICSS, DTS, and CCS simultaneously. The combined model demonstrated satisfactory fit to the data (χ^2^ = 742.38, df = 364, χ^2^/df = 2.04, CFI = 0.962, TLI = 0.955, RMSEA = 0.048, SRMR = 0.042), supporting the adequacy of the overall measurement model. Inter-factor correlations ranged from 0.41 to 0.55, indicating that the constructs were related yet empirically distinct. Following confirmation of the measurement structure, reliability and construct validity for AICSS were assessed. As shown in Table 10, Cronbach’s alpha values ranged from 0.73 to 0.81, and composite reliability coefficients ranged from 0.74 to 0.85, indicating acceptable internal consistency across all dimensions. Average variance extracted values exceeded 0.60 for all four constructs, supporting convergent validity. Discriminant validity was supported, as the square roots of AVE for each dimension were greater than the corresponding inter-factor correlations, which ranged from 0.35 to 0.50.

**Figure 4:**
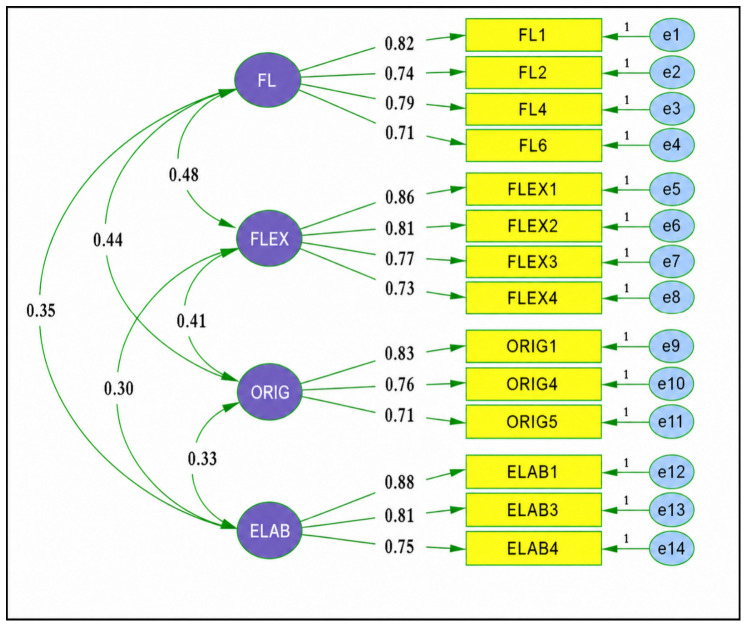
CFA for DTS.

The confirmatory factor model for DTS is presented in Figure 4 and corresponds to the four-dimensional structure identified through exploratory analysis. Standardised factor loadings were substantial across all retained indicators, ranging from 0.71 to 0.82 for Fluency, 0.73 to 0.86 for Flexibility, 0.71 to 0.83 for Originality, and 0.75 to 0.88 for Elaboration. Inter-factor correlations were moderate, with coefficients ranging from 0.30 to 0.48, indicating conceptual relatedness while preserving dimensional distinctiveness within the construct of divergent thinking. The configuration of loadings and latent correlations supports the adequacy of the specified measurement model and is consistent with the scale’s theoretical organisation.

Assessment of reliability and construct validity for DTS followed confirmation of the factor structure. As shown in Table 11, internal consistency was acceptable across dimensions, with Cronbach’s alpha values between 0.71 and 0.79 and composite reliability coefficients ranging from 0.72 to 0.81. Average variance extracted values ranged from 0.59 to 0.66, indicating adequate shared variance at the construct level. Evidence of discriminant validity was observed, as the square roots of the AVEs for each dimension exceeded the corresponding inter-factor correlations (0.28–0.42), supporting differentiation among Fluency, Flexibility, Originality, and Elaboration.

**Figure 5:**
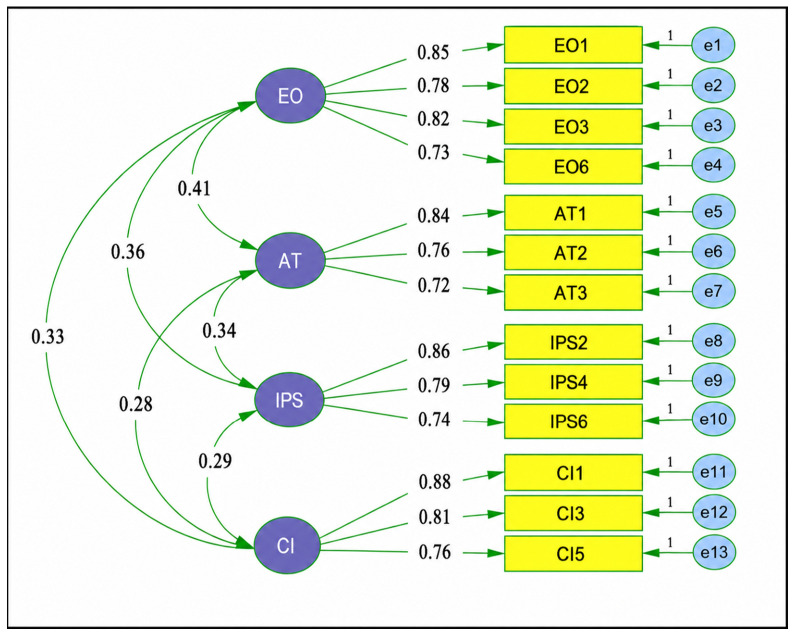
CFA for CSS.

Figure 5 presents the confirmatory factor structure specified for CCS, reflecting the four latent dimensions established at the exploratory stage. Inspection of the standardised solution shows that indicators associated with Expressive Originality loaded between 0.73 and 0.85, while items defining Adaptive Thinking exhibited loadings ranging from 0.72 to 0.84. The Innovative Problem-Solving factor was characterised by coefficients between 0.74 and 0.86. Conceptual Integration was represented by loadings from 0.76 to 0.88. Latent correlations across factors were moderate, varying from 0.28 to 0.41, indicating meaningful associations without compromising dimensional separation. The overall configuration supports the adequacy of the proposed measurement model and is consistent with the conceptualisation of cognitive creativity underlying the scale.

Following confirmation of the CCS measurement structure, reliability and construct validity were examined. As reported in Table 12, Cronbach’s alpha values ranged from 0.72 to 0.80, and composite reliability coefficients ranged from 0.73 to 0.81, indicating satisfactory internal consistency across all four dimensions. Average variance extracted values exceeded 0.60 for Expressive Originality, Adaptive Thinking, Innovative Problem-Solving, and Conceptual Integration, supporting convergent validity. Discriminant validity was evident, as the square roots of AVE for each construct were greater than the corresponding inter-factor correlations, which ranged from 0.24 to 0.37, indicating adequate separation among the dimensions of cognitive creativity.

In addition to the Fornell–Larcker criterion, discriminant validity was further assessed using the heterotrait–monotrait ratio (HTMT). As shown in Table 13, all HTMT values were below the recommended threshold of 0.85 (Henseler et al., 2015), providing additional evidence of discriminant validity among the three constructs.

Measurement invariance across the four AI-experience groups was examined prior to testing the moderated mediation model. Configural, metric, and scalar invariance were evaluated to determine whether the measurement structure operated equivalently across groups. The results are presented in Table 14.

### 4.3. Preliminary Measurement Evaluation

**Figure 6:**
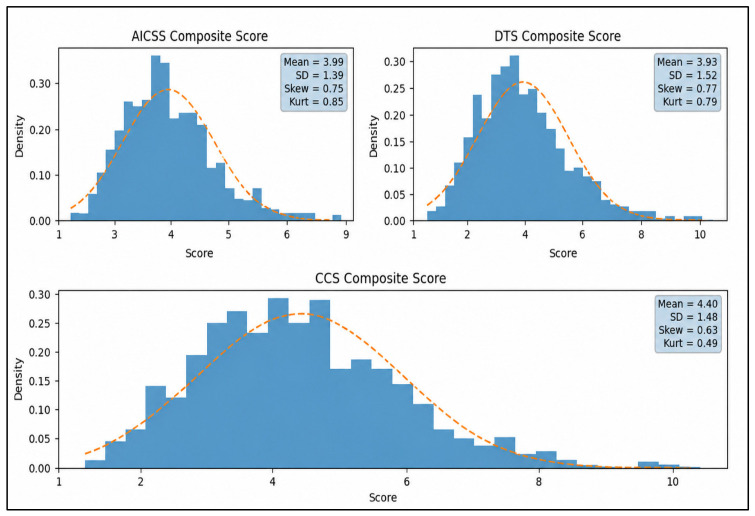
Distribution of Composite Scores for AICSS, DTS, and CCS.

Following CFA, a preliminary measurement evaluation was conducted to assess internal consistency and construct validity prior to structural modelling. This step focused on assessing reliability, convergent validity, and discriminant validity for the retained measurement models. The distributional characteristics of the composite scores were examined to assess the suitability of the data for subsequent structural modelling. As illustrated in Figure 6, the composite scores for AICSS showed a mean of 3.99 (SD = 1.39), with positive skewness (0.75) and kurtosis (0.85), indicating a moderately asymmetric but acceptable distribution. The DTS composite scores had a mean of 3.93 (SD = 1.52), with skewness of 0.77 and kurtosis of 0.79, indicating a similar distribution. For CCS, the composite scores were centred at a higher mean of 4.40 (SD = 1.48), with skewness (0.63) and kurtosis (0.49) values indicating closer approximation to normality. Across all constructs, skewness and kurtosis values remained within acceptable ranges for latent variable modelling, supporting the appropriateness of proceeding with structural analysis.

Following an inspection of the score distributions, collinearity diagnostics were conducted to assess the independence of the predictors prior to structural modelling. As reported in Table 15, variance inflation factor values ranged from 1.36 to 1.94, and tolerance values ranged from 0.52 to 0.74 across all predictors and interaction terms. These values indicate the absence of problematic multicollinearity among AICSS, DTS, duration of AI experience, and the interaction terms, supporting the suitability of the predictor set for subsequent moderated mediation analysis.

### 4.4. Hypotheses Testing

**Figure 7:**
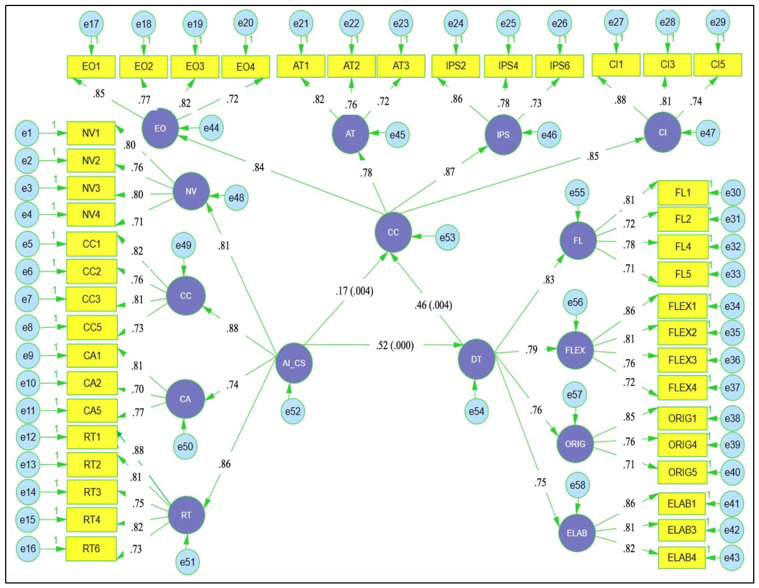
SEM path model.

SEM was employed to test the hypothesised relationships among AI cognitive stimulation, divergent thinking, cognitive creativity, and duration of AI experience. The hypothesised structural model demonstrated good fit to the data, χ^2^ = 759.84, df = 366, χ^2^/df = 2.08, CFI = 0.960, TLI = 0.956, RMSEA = 0.049, and SRMR = 0.044, indicating that the proposed model provided an adequate representation of the observed data. The results of the structural model are summarised in Table 16 and represented in Figure 7. Structural path significance was evaluated using bootstrapped confidence intervals and associated *p*-values; therefore, the reporting focused on standardised coefficients, unstandardized coefficients, and confidence interval estimates rather than critical ratio statistics.

Consistent with H1, AI cognitive stimulation showed a strong positive association with divergent thinking (β = 0.52, *p* < .001), indicating that higher levels of cognitively engaging AI interaction were associated with greater generative ideation. Divergent thinking, in turn, demonstrated a substantial positive relationship with cognitive creativity (β = 0.46, *p* < .001), supporting H2 and indicating that learners’ capacity for flexible and original idea generation was closely linked to creative cognitive outcomes in EFL contexts. AI cognitive stimulation also exhibited a direct relationship with cognitive creativity (β = 0.17, *p* = .004), supporting H3. This direct effect was notably smaller than the indirect pathway operating through divergent thinking. Mediation analysis confirmed H4, with a statistically significant indirect effect of AI cognitive stimulation on cognitive creativity via divergent thinking (β = 0.24, *p* < .001). In line with H5, the magnitude of the indirect effect exceeded that of the direct effect, indicating that divergent thinking constituted the dominant explanatory pathway linking AI-related cognitive stimulation to creative cognition. The moderated mediation hypothesis (H6) was also supported. Duration of AI experience significantly conditioned both the path from AI cognitive stimulation to divergent thinking (β = 0.12, *p* = .006) and the path from divergent thinking to cognitive creativity (β = 0.10, *p* = .009). The index of moderated mediation was statistically significant (β = 0.06, 95% CI [0.02, 0.11]), indicating that the indirect effect of AI cognitive stimulation on cognitive creativity strengthened as learners’ experience with AI increased. This pattern suggests that extended engagement with AI systems was associated with greater cognitive utilisation of AI-generated input rather than simple exposure effects. The model explained 30% of the variance in divergent thinking (R^2^ = 0.30) and 37% of the variance in cognitive creativity (R^2^ = 0.37).

**Figure 8:**
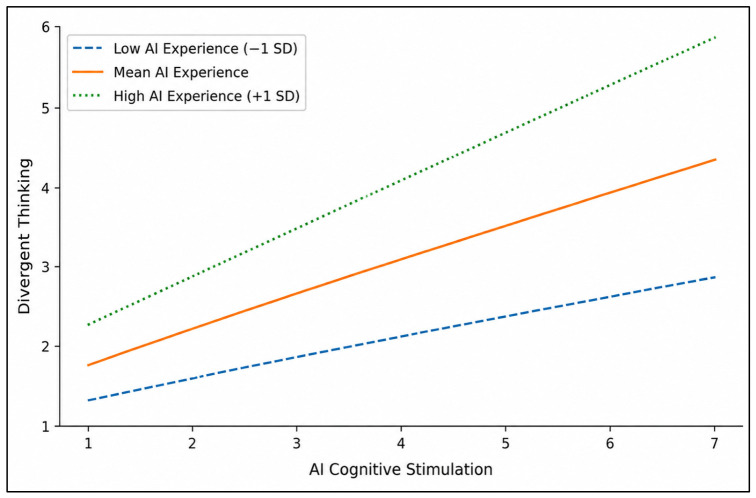
Simple slopes of the moderation effects across combined AI experience.

**Figure 9:**
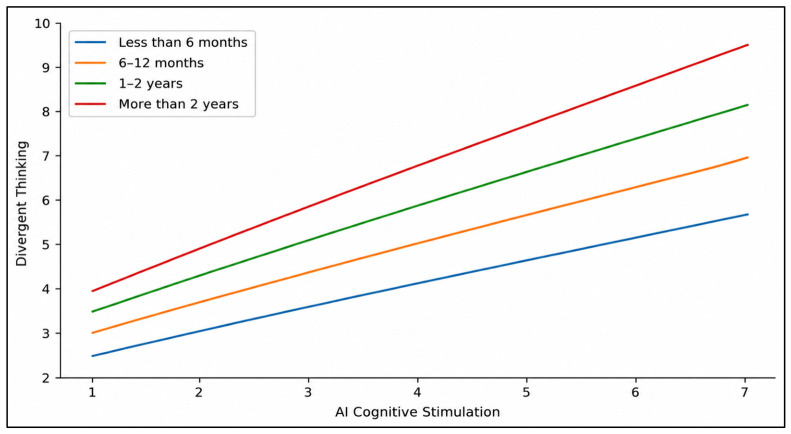
Simple slopes of the moderation effects across different AI experience categories.

To clarify the moderation effect, post-estimation simple slope analyses were conducted to examine the association between AI cognitive stimulation and divergent thinking across levels of AI experience. As shown in Figure 8, the relationship remained positive at low (−1 SD), mean, and high (+1 SD) levels of AI experience, with visibly steeper slopes at higher experience levels. The conditional slope estimates increased from β = 0.31 at low AI experience (−1 SD), to β = 0.44 at the mean level, and to β = 0.57 at high AI experience (+1 SD), indicating progressively stronger associations between AI cognitive stimulation and divergent thinking as AI experience increased. This pattern indicates that the cognitive stimulation associated with AI engagement translated into stronger divergent thinking as learners accumulated greater experience interacting with AI systems. A complementary depiction using categorical experience groups is presented in Figure 9. The plotted trajectories show a monotonic increase in slope strength from learners with fewer than 6 months of AI experience to those with more than 2 years of sustained use. Learners in the highest-experience group showed the steepest increase in divergent thinking as AI cognitive stimulation increased, whereas those with limited experience showed a shallower, though still positive, association. These figures clarify how AI experience conditions the strength of the relationship, illustrating variation in magnitude rather than changes in direction.

## 5. Discussion

### 5.1. Cognitive Pathways Linking AI Cognitive Stimulation and Creative Outcomes (H1, H2, H3)

The findings indicate a clear pattern among the three core constructs. AI cognitive stimulation showed a strong positive association with divergent thinking, which in turn showed a strong positive association with cognitive creativity, while AI cognitive stimulation exhibited a statistically significant but weaker direct association with cognitive creativity. This configuration suggests that cognitively stimulating AI engagement is closely related to learners’ generative ideation processes, and that creative cognition in EFL contexts is more strongly linked to divergent thinking than to AI-related stimulation alone.

This pattern can be explained by the distinct roles played by external stimulation and internal cognitive processing in EFL learners’ cognitive creativity. This interpretation is consistent with the cognitive perspective developed in the literature review, which conceptualises cognitive creativity as arising from semantic exploration, conceptual restructuring, and flexible idea generation rather than from exposure to external stimuli alone. Support for H1 reflects how cognitively demanding AI engagement, defined here by novelty, cognitive challenge, curiosity activation, and reflective processing, broadens associative search and encourages ideational flexibility during second-language use, both core characteristics of divergent thinking (Gu et al., 2022; Johnson et al., 2022). For EFL learners, such stimulation disrupts reliance on familiar lexical and syntactic routines and promotes exploration of alternative expressions and meanings, expanding the range of ideas available during language production. Support for H2 is consistent with evidence indicating that divergent thinking is closely associated with creative language use in an additional language, as the generation, shifting, and elaboration of multiple ideas are linked to flexible semantic organisation under conditions of linguistic constraint (Patterson et al., 2023; Said-Metwaly et al., 2022). In EFL contexts, cognitive creativity depends on learners’ capacity to reorganise meaning across limited linguistic resources, making divergent thinking particularly relevant for producing original and adaptive language solutions rather than reproducing conventional forms. The weaker direct association observed for H3 may reflect the limits of stimulation without substantial internal cognitive engagement in EFL learning. Exposure to alternative linguistic forms or perspectives through AI does not, in itself, yield cognitive creativity unless learners actively evaluate, select, and restructure meaning during second-language processing (Khalil & Moustafa, 2022). Without such regulation, AI-generated input may support surface-level variation in EFL output while leaving deeper ideational organisation and creative cognition essentially unchanged.

Comparison with prior research reinforces this interpretation. Reviews of AI-supported EFL learning note that creativity-related outcomes are often inferred from task products or performance indicators, with limited attention to the cognitive processes through which such outcomes arise (AlTwijri & Alghizzi, 2024; Katsarou et al., 2023; Yang & Kyun, 2022). More recent work suggests that creative gains depend on how learners cognitively engage with AI-generated input rather than on AI use itself, particularly when learners critically transform, revise, and reinterpret AI output (Abdelhalim, 2024; Shi & Aryadoust, 2024). Recent research further indicates that AI-supported language learning is most conducive to higher-order outcomes when learners actively evaluate, adapt, and extend AI-generated content rather than rely on it uncritically (G. L. Liu et al., 2025). The present findings align with this emerging evidence by showing that AI cognitive stimulation is more strongly associated with cognitive creativity when considered alongside divergent thinking, supporting a process-oriented interpretation of the observed associations among AI cognitive stimulation, divergent thinking, and cognitive creativity in AI-supported EFL learning.

### 5.2. Divergent Thinking as the Central Explanatory Mechanism (H4, H5)

Findings related to H4 and H5 indicate that divergent thinking represented the primary pathway through which AI cognitive stimulation was associated with EFL learners’ cognitive creativity. The indirect effect operating through divergent thinking was statistically significant and exceeded the magnitude of the direct association between AI cognitive stimulation and cognitive creativity. This pattern indicates that most of the variance in the association between AI-related cognitive conditions and creative cognition was mediated by learners’ generative ideation processes rather than by AI stimulation alone.

This prominence of the mediated pathway may reflect the role of divergent thinking in organising internal ideas during language use. This finding is consistent with the cognitive framework developed in the literature review, which positions divergent thinking as the central mechanism by which semantic exploration, conceptual restructuring, and flexible idea generation translate into cognitive creativity. Support for H4 indicates that AI cognitive stimulation introduces external variation, whereas divergent thinking governs how this variation is transformed through ideational search, semantic recombination, and internal selection. Cognitive research indicates that creative outcomes depend on how learners navigate semantic memory, particularly their ability to access and integrate more distant associations during ideation (Bai et al., 2021; Ovando-Tellez et al., 2022). For EFL learners, this process is especially consequential because creative language use requires active reorganisation of meaning across limited lexical and syntactic resources. Divergent thinking, therefore, appears to represent the cognitive pattern through which AI-related stimulation is associated with original and adaptive language solutions rather than remaining external input. Support for H5 is consistent with this interpretation. When divergent thinking is engaged, AI cognitive stimulation contributes to creative cognition primarily through internally regulated semantic exploration rather than through direct exposure effects. Neurocognitive accounts of creativity show that creative transformation depends on internal network dynamics that determine whether external input broadens or constrains ideational search (Shofty et al., 2022). In the absence of such regulation, stimulation may increase surface-level variation without producing substantive creative cognition. Evidence from AI-supported learning aligns with this interpretation, showing that extensive reliance on AI dialogue systems can weaken independent reasoning and generative processing when learners defer ideational responsibility to the system (Zhai et al., 2024). The present findings, therefore, suggest that divergent thinking represents the central cognitive pattern underlying the association between AI-related stimulation and EFL learners’ cognitive creativity.

Comparison with prior work at the level of cognitive mechanism further clarifies the contribution of the present findings. Studies examining AI-supported learning increasingly acknowledge that higher-order outcomes depend on patterns of learner engagement. Nevertheless, few have empirically traced how external stimulation is cognitively transformed into creative capacity. Research on AI-supported writing and longitudinal engagement indicates that creative development is predicted by learners’ sustained cognitive involvement and self-directed idea construction rather than by AI assistance alone (Essel et al., 2023; Shen & Teng, 2024). Related evidence from broader educational contexts shows that generative systems may expand idea spaces, but the quality and originality of outcomes depend on learners’ internal regulation of ideational search and selection (Doshi & Hauser, 2023; Ovando-Tellez et al., 2022). In EFL contexts, reviews caution that when learners defer ideational responsibility to AI systems, generative processing and independent reasoning may weaken despite increased exposure to novel input (Zhai et al., 2024). The present findings advance this literature by showing that divergent thinking is the dominant pathway linking AI cognitive stimulation and cognitive creativity, thereby specifying an internal cognitive pattern that previous studies have acknowledged conceptually but left unmodelled. This result reinforces the argument that creative outcomes in AI-supported EFL learning are primarily shaped by learners’ cognitive processing of AI-generated input rather than by exposure to AI systems alone.

Recent research also presents a more complex picture of the relationship between AI and creativity. Studies have shown that generative AI systems can outperform human participants on certain divergent-thinking tasks (Hubert et al., 2024), while AI-assisted idea generation may increase individual creativity yet reduce diversity across groups by encouraging convergence around similar solutions (Doshi & Hauser, 2023). Related work on AI augmentation in creative problem-solving suggests that the benefits of AI depend on how learners engage with and evaluate AI-generated content rather than on AI assistance alone (Boussioux et al., 2024).

### 5.3. AI Experience as a Conditioning Factor in the Cognitive Process (H6)

Findings related to H6 indicate that learners’ duration of AI experience was associated with variation in the indirect relationship between AI cognitive stimulation and cognitive creativity through divergent thinking. The moderated mediation analysis showed that the indirect pathway was modestly but meaningfully conditioned by AI experience, with the association becoming somewhat stronger at higher levels of experience while remaining consistent in direction across experience groups. This result indicates that AI experience was associated with differences in the magnitude of the cognitive pathway linking stimulation, divergent thinking, and creativity, rather than altering the structure of the relationships themselves among EFL learners. This finding is consistent with the conceptual framework proposed in the study, which views AI experience as a condition shaping learners’ cognitive engagement with AI-generated input rather than as a direct source of cognitive creativity.

This pattern may reflect differences in learners’ cognitive orientation toward AI use across varying levels of experience. With limited experience, EFL learners tend to devote substantial cognitive resources to managing system functionality and maintaining task accuracy, thereby restricting ideational expansion and limiting engagement with divergent thinking. As experience increases, interaction with AI becomes more familiar and less cognitively demanding at the operational level, allowing attentional resources to be redirected toward idea generation, comparison, and semantic restructuring. Empirical research on AI adoption in EFL contexts supports this interpretation, showing that the early stages of AI use are characterised by uncertainty and surface-level engagement, whereas extended experience fosters more strategic and reflective interaction with AI-generated content (G. L. Liu et al., 2025; H. Wu et al., 2024). Under these conditions, divergent thinking may become more strongly associated with AI-related cognitive stimulation and cognitive creativity.

Comparison with prior research situates this finding within the broader body of work on AI-supported learning and learner development. Longitudinal studies of AI-supported writing report that higher-order outcomes increasingly depend on learners’ self-directed cognitive engagement rather than on AI assistance as experience accumulates (Shen & Teng, 2024). Related evidence indicates that learners with greater familiarity with generative systems are more likely to treat AI output as provisional material, subject to questioning, revision, and recombination, thereby supporting deeper ideational processing (Essel et al., 2023; Song & Song, 2023). At the same time, reviews caution that prolonged but uncritical reliance on AI dialogue systems may weaken independent reasoning and generative processing, even among experienced users (Zhai et al., 2024). The present findings align with this body of work by showing that AI experience modestly but meaningfully conditions the divergent-thinking pathway associated with cognitive creativity, reinforcing the view that experience is associated with cognitive engagement rather than directly producing creative development in EFL learning.

## 6. Theoretical and Practical Implications

The theoretical contribution of this study follows directly from the structural findings. The results show that AI cognitive stimulation was associated with EFL learners’ cognitive creativity primarily through divergent thinking, while the direct association remained comparatively limited. This pattern provides empirical support for a process-oriented account of creativity in AI-supported EFL learning, in which creative cognition is associated with learners’ internal ideational regulation rather than with exposure to generative systems alone. Modelling divergent thinking as a mediating pathway clarifies the cognitive pattern through which AI-related cognitive conditions are associated with creative capacity under second-language constraints, addressing a long-standing gap in research that has tended to infer creativity from task products or performance indicators. The moderated mediation results further extend this account by showing that learners’ accumulated AI experience was connected with variation in the strength of this cognitive pathway without altering its overall structure. AI experience, therefore, appears to function as a condition associated with the intensity of ideational processing and semantic reorganisation rather than as an independent source of creativity. This theoretical framing repositions creativity in EFL contexts as a cognitively grounded phenomenon associated with learner agency and internal control processes operating within AI-supported environments.

The practical implications follow from the same empirical logic. Since divergent thinking accounted for the dominant pathway linking AI cognitive stimulation to cognitive creativity, instructional uses of AI that rely on presenting model answers, automated feedback, or surface-level variation may be less likely to support creative development in EFL learning. Pedagogical designs should instead require learners to generate alternatives, compare AI-generated possibilities, justify their choices, and restructure meaning in language use. The conditioning role of AI experience suggests that learners in the early stages of AI use benefit from support that reduces operational demands and redirects attention toward ideational work. In contrast, more experienced learners require tasks that sustain responsibility for idea generation and evaluative judgement. For curriculum design and classroom practice, the findings indicate that creative outcomes depend less on the sophistication of AI tools than on whether learning activities channel AI-related stimulation through cognitively demanding, learner-controlled processes. Under these conditions, AI functions as a stimulus for creative cognition rather than as a substitute for it.

## 7. Limitations and Recommendations for Future Research

Several limitations of the present study point to clear directions for future research. First, the study relied on self-report instruments to capture AI cognitive stimulation, divergent thinking, and cognitive creativity, which may reflect learners’ perceptions rather than their actual cognitive behaviour; future research should integrate performance-based tasks, think-aloud protocols, or trace data from AI–learner interactions to capture cognitive processes as they unfold during EFL use. Second, the cross-sectional design restricts claims about causal sequencing among the constructs; longitudinal or intervention-based studies would allow examination of how AI cognitive stimulation, divergent thinking, and creative cognition develop and influence one another over time. Third, divergent thinking was operationalised through a domain-adapted scale rather than task-embedded language activities, which may limit sensitivity to real-time ideational shifts during speaking or writing; subsequent studies should examine divergent thinking within authentic EFL tasks, linking cognitive indicators directly to observable language production. Fourth, AI experience was measured solely by duration, which does not account for qualitative differences in engagement, strategy use, or dependency patterns; the measure also failed to capture potentially important dimensions such as frequency of AI use, depth of interaction, or task-specific purposes for AI engagement. Moreover, participants reported substantially different levels of prior AI experience, ranging from less than 6 months to more than 2 years. Although AI experience was incorporated into the model as a moderating variable, this variation may have introduced additional heterogeneity that was not fully captured by duration alone. Future research should therefore adopt more fine-grained multidimensional measures of AI experience to clarify how different forms of AI engagement shape cognitive and creative processes in AI-supported EFL learning. Fifth, the participant sample consisted of undergraduate EFL learners from a restricted institutional context, which limits transferability; further research should test the proposed model across educational levels, instructional settings, and linguistic environments to assess its robustness beyond the current population. Although common-method bias diagnostics suggested that method-related variance was unlikely to substantially affect the findings, the use of self-report measures collected during a single administration remains a potential source of bias. In addition, AI cognitive stimulation, divergent thinking, and cognitive creativity are conceptually related constructs, and some degree of shared variance may remain despite evidence supporting discriminant validity. Furthermore, participants were recruited through intact classes within universities, and the analyses did not explicitly model potential clustering effects. Future research should therefore employ multi-method, longitudinal, and multilevel designs to examine the robustness of the proposed relationships across different educational and cultural contexts. In addition, the sample showed a gender imbalance, with female participants constituting a larger proportion of the dataset than male participants. Although the study focused on structural relationships rather than gender comparisons, this distribution may limit the representativeness of the findings across broader EFL populations. Future research should therefore examine the proposed model using more demographically balanced samples.

## 8. Conclusions

This study examined how AI cognitive stimulation relates to EFL learners’ cognitive creativity, as measured by divergent thinking, with attention to the conditioning role of AI experience. The results indicate that AI cognitive stimulation was associated with cognitive creativity both directly and indirectly through divergent thinking, and that the strength of this indirect relationship varied with learners’ AI experience, thereby addressing the central research question and supporting all proposed hypotheses. Creative cognition in AI-supported EFL learning appears to depend primarily on internal ideational processes rather than direct exposure to generative systems. Divergent thinking constituted the principal pathway linking cognitively stimulating AI engagement to creative capacity, while accumulated AI experience strengthened this pathway without altering its structure. These results support a process-oriented account of creativity in EFL contexts, positioning learner-controlled semantic exploration and evaluative regulation as central to translating AI-related stimulation into cognitive creativity.

## Figures and Tables

**Table 1:** Demography (N = 878).

Variable	Frequency	Percentage%
**Gender**		
Male	302	34.4
Female	576	65.6
**Age group**		
18–20 years	238	27.1
21–23 years	392	44.7
24–26 years	168	19.1
27 years or above	80	9.1
**Year of study**		
First year	214	24.4
Second year	256	29.2
Third year	238	27.1
Fourth year or above	170	19.4
**Self-rated English proficiency**		
Beginner	186	21.2
Intermediate	472	53.8
Advanced	220	25.1
**Frequency of AI use for learning English**		
Never	64	7.3
Rarely	142	16.2
Sometimes	312	35.5
Often	238	27.1
Always	122	13.9
**Duration of AI experience**		
Less than 6 months	184	21.0
6–12 months	246	28.0
1–2 years	258	29.4
More than 2 years	190	21.6

**Table 2:** KMO and BTS results.

Scale	KMO	Bartlett’s χ^2^	df	*p*
AI Cognitive Stimulation Scale (AICSS)	0.94	8123.45	276	<.001
Divergent Thinking Scale (DTS)	0.92	7648.19	276	<.001
Cognitive Creativity Scale (CCS)	0.93	7896.72	276	<.001

**Table 3:** Total variance explained for AICSS.

Component	Eigenvalue	% of Variance	Cumulative %
1	8.21	34.21	34.21
2	3.42	14.25	48.46
3	2.11	8.79	57.25
4	1.56	6.50	63.75
5	0.92	3.83	67.58
6	0.81	3.38	70.96
7	0.74	3.08	74.04
8	0.69	2.88	76.92
9	0.63	2.63	79.55
10	0.58	2.42	81.97
11	0.54	2.25	84.22
12	0.49	2.04	86.26
13	0.46	1.92	88.18
14	0.42	1.75	89.93
15	0.39	1.63	91.56
16	0.36	1.50	93.06
17	0.33	1.38	94.44
18	0.30	1.25	95.69
19	0.27	1.13	96.82
20	0.24	1.00	97.82
21	0.21	0.88	98.70
22	0.18	0.75	99.45
23	0.09	0.38	99.83
24	0.04	0.17	100.00

**Table 4:** Total variance explained for DTS.

Component	Eigenvalue	% of Variance	Cumulative %
1	7.64	31.83	31.83
2	3.26	13.58	45.41
3	2.34	9.75	55.16
4	1.82	7.58	62.74
5	0.98	4.08	66.82
6	0.87	3.63	70.45
7	0.79	3.29	73.74
8	0.73	3.04	76.78
9	0.68	2.83	79.61
10	0.62	2.58	82.19
11	0.58	2.42	84.61
12	0.53	2.21	86.82
13	0.49	2.04	88.86
14	0.45	1.88	90.74
15	0.41	1.71	92.45
16	0.38	1.58	94.03
17	0.35	1.46	95.49
18	0.32	1.33	96.82
19	0.29	1.21	98.03
20	0.26	1.08	99.11
21	0.19	0.79	99.90
22	0.02	0.08	99.98
23	0.01	0.01	99.99
24	0.00	0.01	100.00

**Table 5:** Total variance explained for CCS.

Component	Eigenvalue	% of Variance	Cumulative %
1	7.92	33.00	33.00
2	3.18	13.25	46.25
3	2.27	9.46	55.71
4	1.68	7.00	62.71
5	0.95	3.96	66.67
6	0.86	3.58	70.25
7	0.78	3.25	73.50
8	0.72	3.00	76.50
9	0.66	2.75	79.25
10	0.61	2.54	81.79
11	0.56	2.33	84.12
12	0.52	2.17	86.29
13	0.48	2.00	88.29
14	0.44	1.83	90.12
15	0.40	1.67	91.79
16	0.37	1.54	93.33
17	0.34	1.42	94.75
18	0.31	1.29	96.04
19	0.28	1.17	97.21
20	0.25	1.04	98.25
21	0.22	0.92	99.17
22	0.17	0.71	99.88
23	0.02	0.08	99.96
24	0.01	0.04	100.00

**Table 6:** Rotated factor loadings for AICSS.

Item	1	2	3	4
NV1	0.84	0.12	0.09	0.05
NV2	0.81	0.14	0.11	0.07
NV3	0.78	0.10	0.13	0.06
NV5	0.73	0.16	0.12	0.08
NV4	0.22	0.18	0.15	0.11
NV6	0.19	0.21	0.17	0.09
CC1	0.11	0.86	0.14	0.10
CC2	0.13	0.83	0.16	0.12
CC3	0.09	0.79	0.18	0.14
CC5	0.15	0.71	0.19	0.13
CC4	0.20	0.27	0.22	0.16
CC6	0.18	0.25	0.24	0.17
CA1	0.12	0.17	0.82	0.14
CA2	0.14	0.19	0.78	0.16
CA5	0.16	0.21	0.71	0.18
CA3	0.23	0.24	0.28	0.20
CA4	0.21	0.26	0.29	0.22
CA6	0.19	0.23	0.25	0.24
RT1	0.10	0.14	0.17	0.88
RT2	0.12	0.16	0.18	0.85
RT3	0.14	0.17	0.19	0.82
RT4	0.15	0.19	0.21	0.78
RT6	0.18	0.22	0.23	0.69
RT5	0.24	0.26	0.27	0.29

**Table 7:** Rotated factor loadings for DTS.

Item	1	2	3	4
FL1	0.86	0.14	0.09	0.07
FL2	0.83	0.16	0.11	0.08
FL4	0.79	0.18	0.12	0.10
FL6	0.72	0.20	0.14	0.11
FL3	0.24	0.22	0.18	0.16
FL5	0.27	0.25	0.21	0.19
FLEX1	0.15	0.88	0.13	0.10
FLEX2	0.17	0.85	0.15	0.12
FLEX3	0.18	0.81	0.17	0.14
FLEX4	0.20	0.76	0.19	0.15
FLEX5	0.26	0.29	0.22	0.18
FLEX6	0.24	0.27	0.25	0.20
ORIG1	0.11	0.14	0.84	0.13
ORIG4	0.13	0.16	0.79	0.15
ORIG5	0.15	0.18	0.73	0.17
ORIG2	0.22	0.24	0.28	0.20
ORIG3	0.25	0.26	0.29	0.22
ORIG6	0.21	0.23	0.27	0.24
ELAB1	0.12	0.15	0.18	0.87
ELAB3	0.14	0.17	0.20	0.83
ELAB4	0.16	0.19	0.22	0.78
ELAB2	0.24	0.26	0.25	0.29
ELAB5	0.23	0.27	0.28	0.26
ELAB6	0.22	0.25	0.27	0.28

**Table 8:** Rotated factor loadings for CCS.

Item	1	2	3	4
EO1	0.87	0.12	0.10	0.08
EO2	0.84	0.14	0.11	0.09
EO4	0.79	0.16	0.13	0.10
EO6	0.72	0.18	0.15	0.12
EO3	0.24	0.22	0.18	0.16
EO5	0.26	0.23	0.20	0.17
AT1	0.14	0.86	0.13	0.11
AT2	0.16	0.82	0.15	0.13
AT3	0.18	0.77	0.17	0.14
AT4	0.25	0.28	0.22	0.19
AT5	0.24	0.27	0.24	0.20
AT6	0.23	0.26	0.25	0.21
IPS2	0.12	0.15	0.85	0.14
IPS4	0.14	0.17	0.80	0.16
IPS6	0.16	0.19	0.74	0.18
IPS1	0.22	0.24	0.29	0.21
IPS3	0.25	0.26	0.28	0.23
IPS5	0.24	0.27	0.26	0.24
CI1	0.11	0.14	0.16	0.88
CI3	0.13	0.16	0.18	0.84
CI5	0.15	0.18	0.20	0.79
CI2	0.23	0.25	0.24	0.29
CI4	0.24	0.26	0.26	0.28
CI6	0.22	0.24	0.27	0.26

**Table 9:** Model fits.

Fit Index	AICSS	DTS	CCS
χ^2^	214.63	198.47	186.92
df	98	94	88
χ^2^/df	2.19	2.11	2.12
TLI	0.958	0.953	0.961
CFI	0.964	0.961	0.968
RMSEA	0.052	0.049	0.047
SRMR	0.041	0.043	0.039

**Table 10:** Convergence and discriminant validity of AICSS.

	Alpha	CR	AVE	1	2	3	4
**1 = NV**	0.75	0.79	0.61	**0.78**			
**2 = CC**	0.80	0.82	0.64	0.50	**0.80**		
**3 = CA**	0.73	0.74	0.61	0.40	0.48	**0.78**	
**4 = RT**	0.81	0.85	0.64	0.35	0.43	0.42	**0.80**

Note. Diagonal values shown in bold represent the square root of the average variance extracted (√AVE). Discriminant validity is supported when the √AVE for each construct exceeds its correlations with other constructs.

**Table 11:** Convergence and discriminant validity of DTS.

	Alpha	CR	AVE	1	2	3	4
**1 = FL**	0.74	0.77	0.59	**0.77**			
**2 = FLEX**	0.78	0.81	0.63	0.41	**0.79**		
**3 = ORIG**	0.71	0.72	0.59	0.42	0.40	**0.77**	
**4 = ELAB**	0.79	0.80	0.66	0.35	0.28	0.30	**0.82**

Note. Diagonal values shown in bold represent the square root of the average variance extracted (√AVE). Discriminant validity is supported when the √AVE for each construct exceeds its correlations with other constructs.

**Table 12:** Convergence and discriminant validity of CSS.

	Alpha	CR	AVE	1	2	3	4
**1 = EO**	0.80	0.81	0.63	**0.80**			
**2 = AT**	0.72	0.73	0.60	0.37	**0.77**		
**3 = IPS**	0.75	0.77	0.64	0.34	0.30	**0.80**	
**4 = CI**	0.78	0.80	0.67	0.31	0.25	0.24	**0.82**

*Note.* Diagonal values shown in bold represent the square root of the average variance extracted (√AVE). Discriminant validity is supported when the √AVE for each construct exceeds its correlations with other constructs.

**Table 13:** HTMT ratios for discriminant validity assessment.

Higher-Order Construct	1	2	3
1 = AICSS	1.0		
2 = CCS	0.48	1.00	
3 = DTS	0.59	0.62	1.00

**Table 14:** Measurement invariance across AI-experience groups.

Model	χ^2^/df	CFI	RMSEA	SRMR
**Configural**	2.09	0.958	0.050	0.047
**Metric**	2.00	0.957	0.048	0.052
**Scalar**	1.93	0.956	0.046	0.055

Note. Metric invariance was evaluated relative to the configural model (ΔCFI = −0.001, ΔRMSEA = −0.002, ΔSRMR = 0.005, Δχ^2^(120) = 118.118, *p* = .531). Scalar invariance was evaluated relative to the metric model (ΔCFI = −0.001, ΔRMSEA = −0.002, ΔSRMR = 0.003, Δχ^2^(129) = 132.764, *p* = .393). The changes in CFI, RMSEA, and SRMR remained within recommended thresholds, supporting metric and scalar invariance across AI-experience groups.

**Table 15:** Collinearity test.

Predictor Variable	VIF	Tolerance
AICSS	1.82	0.55
DTS	1.94	0.52
Duration of AI Experience	1.36	0.74
AICSS × AI Experience	1.68	0.60
DTS × AI Experience	1.59	0.63

**Table 16:** SEM path estimates

Path	Unstd. Est (B)	S.E.	Std. Est. (β)	95% CI	*p*
AICSS → DTS	0.56	0.06	0.52	[0.44, 0.68]	<.001
DTS → CCS	0.48	0.05	0.46	[0.38, 0.58]	<.001
AICSS → CCS	0.19	0.07	0.17	[0.06, 0.33]	.004
AICSS → DTS → CCS	0.27	0.04	0.24	[0.18, 0.33]	<.001
AICSS × AI Experience → DTS	0.14	0.05	0.12	[0.04, 0.24]	.006
DTS × AI Experience → CCS	0.11	0.04	0.10	[0.03, 0.19]	.009

## Data Availability

The dataset for this study is available on reasonable request.

## Supplementary Materials

The following supporting information can be downloaded at: https://www.mdpi.com/article/10.3390/jintelligence14070139/s1.
