# Supplementary material for: Modelling Divergent Thinking as a Mediator Between Artificial Intelligence Cognitive Stimulation and EFL Learners’ Cognitive Creativity
Source: J Intell. 2026 Jul 5;14(7):139. doi: 10.3390/jintelligence14070139 (PMC13413378; doi:10.3390/jintelligence14070139)
Supplement: Supplementary file 1 [file jintelligence-14-00139-s001.zip › jintelligence-4248789-supplementary.pdf]

Supplementary S1

Scales Before validation

Demographic Information

Variable	Response Options / Format
**Gender**	☐ Male ☐ Female
**Age**	☐ 18–20 ☐ 21–23 ☐ 24–26 ☐ 27 and above
**University Level**	☐ First Year ☐ Second Year ☐ Third Year ☐ Fourth Year
**Self-Rated English Proficiency (CEFR-based)**	☐ A2 (Pre-Intermediate) ☐ B1 (Intermediate) ☐ B2 (Upper-Intermediate) ☐ C1 (Advanced)
**Frequency of AI Tool Use**	☐ Rarely ☐ Sometimes ☐ Often ☐ Very Often
**Duration of AI Experience**	☐ Less than 6 months ☐ 6–12 months ☐ 1–2 years ☐ More than 2 years

AI Cognitive Stimulation Scale (AICSS)

Dimension / Item	Strongly Disagree	Disagree	Neutral	Agree	Strongly Agree
**Dimension 1 – Novelty and Surprise**					
1. The AI tasks present ideas or examples that feel entirely new to me.	☐	☐	☐	☐	☐
2. I often encounter unexpected language patterns or expressions from AI responses.	☐	☐	☐	☐	☐
3. The content generated by AI makes me see English from a different angle.	☐	☐	☐	☐	☐
4. AI activities introduce perspectives I have never considered before.	☐	☐	☐	☐	☐
5. Working with AI adds an element of surprise to my learning experience.	☐	☐	☐	☐	☐
6. AI-generated input often presents ideas that differ from what I previously believed about language.	☐	☐	☐	☐	☐
**Dimension 2 – Cognitive Challenge**					
7. AI tasks push me to think carefully about how and why words are used.	☐	☐	☐	☐	☐

8. I need to analyse and evaluate AI feedback before deciding what to accept.	☐	☐	☐	☐	☐
9. The AI's suggestions make me compare multiple ways of expressing an idea.	☐	☐	☐	☐	☐
10. Interacting with AI requires sustained mental effort to make sense of its reasoning.	☐	☐	☐	☐	☐
11. The complexity of AI responses encourages me to engage in problem-solving.	☐	☐	☐	☐	☐
12. I find myself thinking harder when completing AI-supported assignments.	☐	☐	☐	☐	☐
**Dimension 3 – Curiosity Activation**					
13. AI-based activities make me want to explore new topics in English.	☐	☐	☐	☐	☐
14. I feel curious to know how AI produces its responses.	☐	☐	☐	☐	☐
15. The AI's output often leads me to search for additional explanations or examples.	☐	☐	☐	☐	☐
16. I become more interested in discovering how language actually works.	☐	☐	☐	☐	☐
17. I am motivated to experiment with AI prompts to see different results.	☐	☐	☐	☐	☐
18. AI-generated responses often trigger questions that extend beyond the topic being studied.	☐	☐	☐	☐	☐
**Dimension 4 – Reflective Thinking**					
19. AI feedback makes me rethink how I learn English effectively.	☐	☐	☐	☐	☐
20. I reflect on my previous errors when comparing them with AI suggestions.	☐	☐	☐	☐	☐
21. Interacting with AI encourages me to question my own assumptions about language use.	☐	☐	☐	☐	☐
22. I use AI responses to assess my current level of understanding.	☐	☐	☐	☐	☐
23. AI-assisted tasks help me notice gaps in my language knowledge.	☐	☐	☐	☐	☐
24. The AI experience pushes me to reconsider my learning strategies.	☐	☐	☐	☐	☐

Divergent Thinking Scale (DTS)

Dimension / Item	Strongly Disagree	Disagree	Neutral	Agree	Strongly Agree
**Dimension 1 – Fluency**					
1. I can generate many ideas when working on English tasks.	☐	☐	☐	☐	☐
2. I usually produce several sentences to express one idea.	☐	☐	☐	☐	☐
3. I can list many examples when discussing a topic in English.	☐	☐	☐	☐	☐
4. I easily come up with alternative phrases or expressions.	☐	☐	☐	☐	☐
5. Ideas come to me quickly when I start brainstorming.	☐	☐	☐	☐	☐
6. I can produce a long series of related ideas without difficulty.	☐	☐	☐	☐	☐
**Dimension 2 – Flexibility**					
7. I can shift to a new approach when one idea does not work.	☐	☐	☐	☐	☐
8. I can view a topic from different perspectives when using English.	☐	☐	☐	☐	☐
9. I can adjust my thinking style to fit the communication context.	☐	☐	☐	☐	☐
10. I easily switch between different ways of reasoning about a problem.	☐	☐	☐	☐	☐
11. I can adapt to different types of English tasks without difficulty.	☐	☐	☐	☐	☐
12. I can reorganise my ideas quickly when needed.	☐	☐	☐	☐	☐
**Dimension 3 – Originality**					
13. I often create ideas that differ from those of my classmates.	☐	☐	☐	☐	☐
14. I enjoy producing expressions that sound new or unusual.	☐	☐	☐	☐	☐

15. I like combining unrelated words or concepts to make new meanings.	☐	☐	☐	☐	☐
16. I prefer presenting my ideas in a unique or creative way.	☐	☐	☐	☐	☐
17. I generate examples that others usually do not think of.	☐	☐	☐	☐	☐
18. I find original solutions to language-related tasks.	☐	☐	☐	☐	☐
**Dimension 4 – Elaboration**					
19. I can expand a short idea into a detailed explanation.	☐	☐	☐	☐	☐
20. I add examples to make my ideas clearer.	☐	☐	☐	☐	☐
21. I develop each idea step by step until it becomes well-structured.	☐	☐	☐	☐	☐
22. I include supporting details to strengthen my explanations.	☐	☐	☐	☐	☐
23. I refine my initial thoughts to make them more coherent.	☐	☐	☐	☐	☐
24. I link related ideas together to build a complete response.	☐	☐	☐	☐	☐

Cognitive Creativity Scale (CCS)

Dimension / Item	Strongly Disagree	Disagree	Neutral	Agree	Strongly Agree
**Dimension 1 – Expressive Originality**					
1. I express my ideas in ways that sound new and distinctive in English.	☐	☐	☐	☐	☐
2. My written or spoken English shows my personal creative style.	☐	☐	☐	☐	☐
3. I can apply new ideas effectively when completing English tasks.	☐	☐	☐	☐	☐
4. My work in English often includes original examples or viewpoints.	☐	☐	☐	☐	☐
5. I use creative expressions that make my ideas memorable.	☐	☐	☐	☐	☐

6. I can transform ordinary topics into interesting new forms.	☐	☐	☐	☐	☐
**Dimension 2 – Adaptive Thinking**					
7. I can modify my ideas when a task requires a different approach.	☐	☐	☐	☐	☐
8. I adapt my learning strategies to suit new English contexts.	☐	☐	☐	☐	☐
9. I can reorganise my work when I discover a better way to express an idea.	☐	☐	☐	☐	☐
10. I combine earlier knowledge with new insights to improve my output.	☐	☐	☐	☐	☐
11. I can transfer what I learn in one task to solve a different problem.	☐	☐	☐	☐	☐
12. I adjust my creative approach depending on the audience or situation.	☐	☐	☐	☐	☐
**Dimension 3 – Innovative Problem-Solving**					
13. I can find inventive solutions when facing communication problems.	☐	☐	☐	☐	☐
14. I use creative methods to handle complex English tasks.	☐	☐	☐	☐	☐
15. I turn language difficulties into opportunities to think creatively.	☐	☐	☐	☐	☐
16. I experiment with different strategies until I solve a problem.	☐	☐	☐	☐	☐
17. I improve my English performance through creative problem-solving.	☐	☐	☐	☐	☐
18. I often discover unexpected ways to fix errors or misunderstandings.	☐	☐	☐	☐	☐
**Dimension 4 – Conceptual Integration**					
19. I link ideas from different topics to form new insights.	☐	☐	☐	☐	☐
20. I combine knowledge from various subjects when using English.	☐	☐	☐	☐	☐
21. I can connect unrelated concepts to express complex ideas.	☐	☐	☐	☐	☐

22. I use analogies or metaphors to explain difficult concepts creatively.	☐	☐	☐	☐	☐
23. I merge ideas from different areas to develop new perspectives.	☐	☐	☐	☐	☐
24. I enjoy discovering relationships between seemingly unrelated ideas.	☐	☐	☐	☐	☐

Scales After validation

Demographic Information

Variable	Response Options / Format
**Gender**	☐ Male ☐ Female
**Age**	☐ 18–20 ☐ 21–23 ☐ 24–26 ☐ 27 and above
**University Level**	☐ First Year ☐ Second Year ☐ Third Year ☐ Fourth Year
**Self-Rated English Proficiency (CEFR-based)**	☐ A2 (Pre-Intermediate) ☐ B1 (Intermediate) ☐ B2 (Upper-Intermediate) ☐ C1 (Advanced)
**Frequency of AI Tool Use**	☐ Rarely ☐ Sometimes ☐ Often ☐ Very Often
**Duration of AI Experience**	☐ Less than 6 months ☐ 6–12 months ☐ 1–2 years ☐ More than 2 years

AI Cognitive Stimulation Scale (AICSS)

Dimension / Item	Strongly Disagree	Disagree	Neutral	Agree	Strongly Agree
**Dimension 1 – Novelty and Surprise**					
1. The AI tasks present ideas or examples that feel entirely new to me.	☐	☐	☐	☐	☐
2. I often encounter unexpected language patterns or expressions from AI responses.	☐	☐	☐	☐	☐
3. The content generated by AI makes me see English from a different angle.	☐	☐	☐	☐	☐
4. Working with AI adds an element of surprise to my learning experience.	☐	☐	☐	☐	☐

**Dimension 2 – Cognitive Challenge**					
5. AI tasks push me to think carefully about how and why words are used.	☐	☐	☐	☐	☐
6. I need to analyse and evaluate AI feedback before deciding what to accept.	☐	☐	☐	☐	☐
7. The AI's suggestions make me compare multiple ways of expressing an idea.	☐	☐	☐	☐	☐
8. The complexity of AI responses encourages me to engage in problem-solving.	☐	☐	☐	☐	☐
**Dimension 3 – Curiosity Activation**					
9. AI-based activities make me want to explore new topics in English.	☐	☐	☐	☐	☐
10. I feel curious to know how AI produces its responses.	☐	☐	☐	☐	☐
11. I am motivated to experiment with AI prompts to see different results.	☐	☐	☐	☐	☐
12. AI-generated responses often trigger questions that extend beyond the topic being studied.	☐	☐	☐	☐	☐
**Dimension 4 – Reflective Thinking**					
13. AI feedback makes me rethink how I learn English effectively.	☐	☐	☐	☐	☐
14. I reflect on my previous errors when comparing them with AI suggestions.	☐	☐	☐	☐	☐
15. Interacting with AI encourages me to question my own assumptions about language use.	☐	☐	☐	☐	☐
16. I use AI responses to assess my current level of understanding.	☐	☐	☐	☐	☐
17. The AI experience pushes me to reconsider my learning strategies.	☐	☐	☐	☐	☐

Divergent Thinking Scale (DTS)

**Dimension / Item**	**Strongly Disagree**	**Disagree**	**Neutral**	**Agree**	**Strongly Agree**
**Dimension 1 – Fluency**					
1. I can generate many ideas when working on English tasks.	☐	☐	☐	☐	☐

2. I usually produce several sentences to express one idea.	☐	☐	☐	☐	☐
3. I easily come up with alternative phrases or expressions.	☐	☐	☐	☐	☐
4. I can produce a long series of related ideas without difficulty.	☐	☐	☐	☐	☐
**Dimension 2 – Flexibility**					
5. I can shift to a new approach when one idea does not work.	☐	☐	☐	☐	☐
6. I can view a topic from different perspectives when using English.	☐	☐	☐	☐	☐
7. I can adjust my thinking style to fit the communication context.	☐	☐	☐	☐	☐
8. I easily switch between different ways of reasoning about a problem.	☐	☐	☐	☐	☐
**Dimension 3 – Originality**					
9. I often create ideas that differ from those of my classmates.	☐	☐	☐	☐	☐
10. I prefer presenting my ideas in a unique or creative way.	☐	☐	☐	☐	☐
11. I generate examples that others usually do not think of.	☐	☐	☐	☐	☐
**Dimension 4 – Elaboration**					
12. I can expand a short idea into a detailed explanation.	☐	☐	☐	☐	☐
13. I develop each idea step by step until it becomes well-structured.	☐	☐	☐	☐	☐
14. I include supporting details to strengthen my explanations.	☐	☐	☐	☐	☐

Cognitive Creativity Scale (CCS)

**Dimension / Item**	**Strongly Disagree**	**Disagree**	**Neutral**	**Agree**	**Strongly Agree**
**Dimension 1 – Expressive Originality**					

1. I express my ideas in ways that sound new and distinctive in English.	☐	☐	☐	☐	☐
2. My written or spoken English shows my personal creative style.	☐	☐	☐	☐	☐
3. I can apply new ideas effectively when completing English tasks.	☐	☐	☐	☐	☐
4. I can transform ordinary topics into interesting new forms.	☐	☐	☐	☐	☐
**Dimension 2 – Adaptive Thinking**					
5. I can modify my ideas when a task requires a different approach.	☐	☐	☐	☐	☐
6. I adapt my learning strategies to suit new English contexts.	☐	☐	☐	☐	☐
7. I can reorganise my work when I discover a better way to express an idea.	☐	☐	☐	☐	☐
**Dimension 3 – Innovative Problem-Solving**					
8. I use creative methods to handle complex English tasks.	☐	☐	☐	☐	☐
9. I improve my English performance through creative problem-solving.	☐	☐	☐	☐	☐
10. I often discover unexpected ways to fix errors or misunderstandings.	☐	☐	☐	☐	☐
**Dimension 4 – Conceptual Integration**					
11. I link ideas from different topics to form new insights.	☐	☐	☐	☐	☐
12. I can connect unrelated concepts to express complex ideas.	☐	☐	☐	☐	☐
13. I merge ideas from different areas to develop new perspectives.	☐	☐	☐	☐	☐
